# OsbHLH064, an IVb bHLH Transcription Factor, Regulates Iron Homeostasis and Enhances Grain Fe Accumulation in Rice

**DOI:** 10.1111/pbi.70593

**Published:** 2026-02-23

**Authors:** Fei Gao, Zhikai Zhu, Kaixin Xue, Zijing Fu, Jitong Yue, Na Zhang, Hongli Zhou, Yaqin Deng, Jing Zhou, Liuhui Kuang, Tao Yan, Lin Li, Christian Dubos, Dezhi Wu

**Affiliations:** ^1^ College of Agronomy Hunan Agricultural University Changsha China; ^2^ Yuelushan Laboratory Changsha China; ^3^ College of Bioscience and Biotechnology Hunan Agricultural University Changsha China; ^4^ IPSiM, CNRS, INRAE, Institut Agro University of Montpellier Montpellier France

**Keywords:** bHLH, iron homeostasis, *Oryza sativa*, transcription factor

## Abstract

Iron (Fe) is an essential micronutrient for plant growth and development. The maintenance of Fe homeostasis relies on sophisticated regulatory networks where bHLH transcription factors play a key role. However, how these factors coordinate to regulate this vital process is not fully understood. Here, we characterise OsbHLH064, a previously unstudied IVb bHLH transcription factor, and reveal its critical role in iron homeostasis in rice. Loss of *OsbHLH064* results in constitutive activation of Fe homeostasis‐related genes under Fe‐sufficient conditions, whereas its overexpression strongly suppresses their expression. Remarkably, *OsbHLH064* overexpression leads to excessive Fe accumulation in roots, shoots and brown rice. Under Fe deficiency, it also triggers ROS overproduction, highlighting its essential role in balancing Fe homeostasis and oxidative stress. Mechanistically, OsbHLH064 forms heterodimers with IVc bHLH transcription factors, and these interactions facilitate its nuclear localisation. OsbHLH064 represses *OsIRO2* and *OsIRO3* by competing with or sequestering IVc activators at shared promoters, thereby limiting downstream transcriptional activation. Furthermore, OsbHLH064 directly binds not only canonical IVb/IVc targets but also a broader set of genes involved in Fe uptake, translocation and signalling. Collectively, these findings establish OsbHLH064 as a central upstream regulator integrating multiple pathways to maintain Fe homeostasis and suggest its potential as a target for biofortification strategies aimed at enhancing iron content in rice grains.

## 1 Introduction

Iron (Fe) is an essential element for nearly all living organisms on Earth. Which is, for instance, because Fe is the cofactor for several metalloproteins that carry out electron transfer functions (Connorton et al. 2017). In plants, Fe is involved in various physiological and biochemical pathways, including respiration and photosynthesis, as well as nitrogen fixation, DNA synthesis and other energy transfer reactions (Briat et al. 2015). Although Fe abundance is high in most soils, its low bioavailability limits its utilisation by plants, especially at neutral to alkaline pH (Guerinot and Yi 1994). In such soils, Fe deficiency becomes one of the limiting factors for plant growth and development, which subsequently alters crop productivity and the quality of their derived products (Briat et al. 2015). However, in certain growth conditions, such as waterlogging, plants also suffer from Fe overload that is toxic for plant cells due to the generation of reactive hydroxyl radicals through the Fenton reactions (Halliwell and Gutteridge 1992). Therefore, Fe levels in plant cells must be tightly regulated.

Fe uptake is the first critical step for maintaining intracellular Fe homeostasis (Gao and Dubos 2021). For this purpose, plants have evolved two distinct Fe uptake strategies (Marschner et al. 1986). Non‐graminaceous plants employ the reduction‐based strategy, also called Strategy I (Kobayashi and Nishizawa 2012). By contrast, graminaceous plants employ the chelation‐based strategy (Strategy II), which involves the biosynthesis and the secretion of high‐affinity Fe chelators known as phytosiderophores of the mugineic acid (MA) family (Kobayashi and Nishizawa 2012; Kobayashi et al. 2014). MAs are secreted into the rhizosphere by the efflux TRANSPORTER OF MAs 1 (OsTOM1) (Nozoye et al. 2011), and the formed Fe(III)‐MA complexes are directly taken up into plant roots by the YELLOW STRIPE‐LIKE 15 (OsYSL15) transporter (Inoue et al. 2009). Interestingly, if under upland soil conditions, the acquisition of Fe(III) in rice mainly relies on the secretion of MAs; under waterlogged soil conditions, rice directly acquires Fe(II) from the soil through the activity of two Fe transporters, namely IRON‐REGULATED TRANSPORTER 1 and 2 (OsIRT1 and 2) (Ishimaru et al. 2006; Nakanishi et al. 2006; Yue et al. 2025).

Despite the differences in Fe uptake strategies between grasses and non‐grass species, accumulating evidence suggests that the core transcriptional regulatory network controlling Fe homeostasis is evolutionarily conserved across these plant lineages. Recent studies have highlighted the complexity of Fe homeostasis regulation, identifying basic helix‐loop‐helix (bHLH) transcription factors as central regulators in this process (Liang 2022; Gao and Dubos 2021; Gao et al. 2019; Riaz and Guerinot 2021; Gao, Robe, and Dubos 2020; Gao, Robe, Bettembourg, et al. 2020). In rice, the bHLH protein OsFIT/OsbHLH156 (FER‐LIKE IRON DEFICIENCY INDUCED TRANSCRIPTION FACTOR) physically interacts with OsIRO2/OsbHLH056 (IRON‐RELATED TRANSCRIPTION FACTOR 2), facilitating its nuclear accumulation and thereby activating the expression of Strategy II genes such as *OsNAS1*, *OsNAS2*, *OsNAAT1*, *OsDMAS1* and *OsYSL15* (Liang et al. 2020; Wang, Li, et al. 2020). Interestingly, OsFIT has also been shown to positively regulate *OsIRT1*, suggesting that OsFIT may act as a regulatory hub connecting both Strategy I‐ and Strategy II‐related pathways in rice (Liang et al. 2020). The expression of *OsFIT* and *OsIRO2* is strongly induced under Fe‐deficient conditions (Liang et al. 2020; Wang, Li, et al. 2020), and members of the bHLH IVc subgroup have emerged as potential candidates to regulate *OsFIT* and *OsIRO2* expression. For instance, it has been shown that OsPRI1/OsbHLH060, OsPRI2/OsbHLH058 and OsPRI3/OsbHLH059 (POSITIVE REGULATOR OF IRON HOMEOSTASIS 1, 2 and 3), three bHLH IVc, directly bind and regulate the expression of *OsIRO2* and *OsIRO3/OsbHLH63*, thereby playing a positive role in mediating the Fe deficiency response (Zhang et al. 2017, 2020; Kobayashi et al. 2019). Additionally, OsbHLH057 (also referred to as OsPRI4) is proposed to contribute positively to the regulation of Fe homeostasis, at least under Fe‐sufficient conditions (Wang, Shinwari, et al. 2022).

By contrast, rice bHLHs from subgroup IVb act as negative regulators of Fe homeostasis. OsIRO3, a representative IVb member, physically interacts with IVc bHLH proteins such as OsPRI1 and OsPRI2, thereby antagonising their transcriptional activation of downstream target genes, thus forming a negative feedback loop that fine‐tunes the Fe deficiency response (Li, Li, et al. 2022; Wang, Ye, et al. 2020). Knockout of *OsIRO3* leads to hypersensitivity to Fe deficiency, characterised by excessive Fe accumulation in shoots, ROS overproduction and progressive necrotic lesions in young leaves under Fe‐deficient conditions (Wang, Itai, et al. 2020; Li, Li, et al. 2022; Wang, Ye, et al. 2020; Zheng et al. 2010). The expression of Fe deficiency inducible genes, including *OsIRO2* and *OsFIT* and their downstream targets, is upregulated in *iro3* loss‐of‐function mutant when compared to the wild type (WT) under both control and early Fe deficiency conditions, whereas their expression levels converged to those of the WT at later stages of Fe deficiency, possibly reflecting feedback repression or other regulatory mechanisms (Wang, Itai, et al. 2020; Li, Li, et al. 2022; Wang, Ye, et al. 2020). Similarly, OsbHLH061 and OsbHLH062, two closely related IVb bHLHs, have also been shown to be implicated in the negative regulation of Fe homeostasis by forming heterodimers with IVc bHLHs (Wang et al. 2025; Wang, Ye, et al. 2022; Li et al. 2025). These IVb bHLHs recruit TOPLESS/TOPLESS‐RELATED (OsTPL/OsTPRs) co‐repressors through their ETHYLENE‐RESPONSIVE ELEMENT BINDING FACTOR‐ASSOCIATED AMPHIPHILIC REPRESSION (EAR) motifs, thereby suppressing the transcriptional activity of IVc bHLHs (Wang et al. 2025; Wang, Ye, et al. 2022; Li et al. 2025). Moreover, OsbHLH062 and OsIRO3 act as cofactors of the E3 ubiquitin ligase OsHRZ1 (HAEMERYTHRIN MOTIF‐CONTAINING REALLY INTERESTING NEW GENE (RING) AND ZINC‐FINGER PROTEIN 1), promoting OsPRI proteins' destabilisation and further repressing Fe signalling (Li et al. 2025). These findings highlight the multifaceted roles of IVb bHLHs in integrating both transcriptional repression and post‐translational regulation to fine‐tune Fe homeostasis.

Among the four IVb subgroup bHLH transcription factors, very little is known about OsbHLH064's potential role in regulating Fe homeostasis. Herein, we revealed that OsbHLH064 is a novel regulator of Fe homeostasis in rice. Loss‐of‐function of *OsbHLH06*4 induces the expression of Fe homeostasis‐related genes under Fe‐sufficient conditions, whereas its overexpression leads to opposite trends. OsbHLH064 forms heterodimers with all IVc bHLHs and was found localised in both the nucleus and the endoplasmic reticulum. OsbHLH064 nuclear accumulation is promoted by its interaction with OsPRIs. OsbHLH064 directly binds to the promoters of a set of Fe homeostasis genes via *E‐box* or *G‐box* motifs, sharing binding sites with OsPRI1. Taken together, these data suggest that OsbHLH064 likely regulates the expression of its target genes, including *OsIRO2* and *OsIRO3*, by competing with or sequestering IVc bHLHs at shared promoter binding sites, thereby limiting downstream gene activation.

## 2 Results

### 2.1 OsbHLH064 Interacts with IVc bHLH Transcription Factors

**FIGURE 1:**
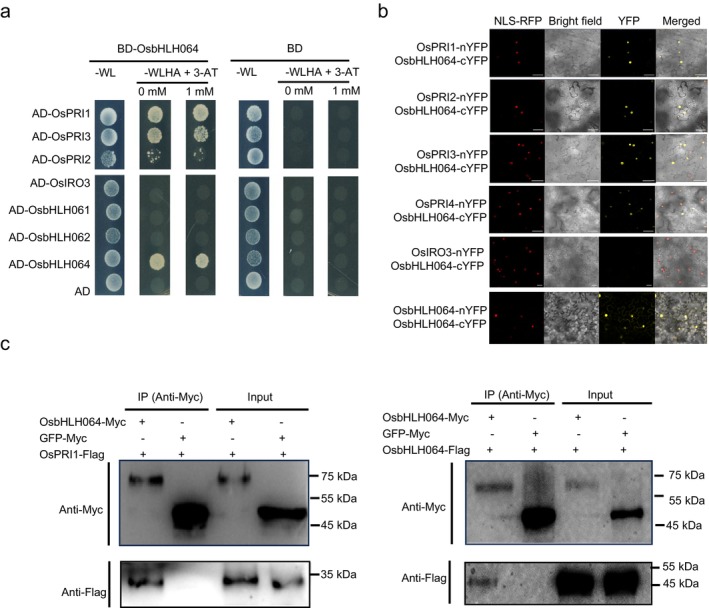
OsbHLH064 forms homodimers and heterodimers with bHLH IVc transcription factors. (a) Yeast two‐hybrid (Y2H) assay showed that OsbHLH064 interacts with itself and with bHLH IVc family members (i.e., OsPRI1, OsPRI2 and OsPRI3), but not with bHLH IVb family members (i.e., OsIRO3, OsbHLH61 and OsbHLH62). Yeast cells co‐expressing BD and AD fusion constructs were selected on SD/‐Leu/‐Trp. Positive interactions are indicated by yeast growth on SD/‐Leu/‐Trp/‐His/‐Ade medium supplemented with 3‐AT. (b) Bimolecular fluorescence complementation (BiFC) assay showing nuclear interactions between OsbHLH064 and OsPRI1, OsPRI2, OsPRI3, OsPRI4 and itself in *N. benthamiana* leaf epidermal cells. YFP fluorescence indicates reconstituted interaction signals, co‐localised with a nuclear‐localised RFP marker (RFP‐NLS) stably expressed in the background line. Scale bars = 50 μm. (c) Co‐immunoprecipitation (Co‐IP) assay showing that OsbHLH064 interacts with OsPRI1 and itself in *N. benthamiana*. Total protein extracts were immunoprecipitated with anti‐Myc antibody and immunoblotted with anti‐Myc and anti‐Flag antibodies. Input and IP fractions are shown.

Homo‐ and hetero‐dimerisations play critical roles in regulating the function of bHLH transcription factors (Gao and Dubos 2024). OsIRO3, OsbHLH61 and OsbHLH62, three rice IVb bHLHs, are known to interact with IVc bHLHs to regulate the expression of downstream genes involved in iron homeostasis (Wang, Ye, et al. 2022; Li et al. 2025; Wang et al. 2025; Wang, Itai, et al. 2020; Li, Li, et al. 2022; Wang, Ye, et al. 2020). To investigate whether OsbHLH064, the fourth member of IVb bHLH subgroup, forms homo‐ or heterodimers with IVc and/or IVb bHLHs, yeast two‐hybrid (Y2H) assays were performed. We found that OsbHLH064 could interact with bHLHs from subgroup IVc (i.e., OsPRI1, OsPRI2 and OsPRI3), but not with those from subgroup IVb (i.e., OsIRO3, OsbHLH061 and OsbHLH062) except with itself (Figure 1a). It is noteworthy that the interaction between OsbHLH064 and OsPRI4 was not determined due to the strong toxicity of OsPRI4 expression in Y2Hgold yeast. To corroborate these protein–protein interactions *in planta*, bimolecular fluorescence complementation (BiFC) assays were performed in *Nicotiana benthamiana* leaves. Co‐expression of OsbHLH064‐nYFP with OsPRI1‐, OsPRI2‐, OsPRI3 and OsPRI4‐cYFP resulted in reconstituted YFP fluorescence signals in the nucleus of *N. benthamiana* epidermal cells (Figure 1b, Figure S1). Co‐expression of OsbHLH064‐nYFP and OsbHLH064‐cYFP also resulted in reconstituted YFP signals in nucleus and cytoplasmic region(Figure 1b, Figure S1). To further confirm these interactions *in planta*, co‐immunoprecipitation (Co‐IP) assays were carried out in tobacco leaves co‐expressing OsbHLH064‐Myc with either OsPRI1‐Flag or OsbHLH064‐Flag. Immunoblot analysis demonstrated that OsbHLH064 co‐precipitated with both OsPRI1 and itself (Figure 1c). Taken together, these results support that OsbHLH064 can form both homodimers and heterodimers with subgroup IVc bHLH transcription factors. It also suggests that through these interactions, OsbHLH064 might modulate bHLH IVc‐mediated transcriptional regulation of Fe homeostasis.

### 2.2 Fe Availability Modulates *OsbHLH064* Expression in Roots

**FIGURE 2:**
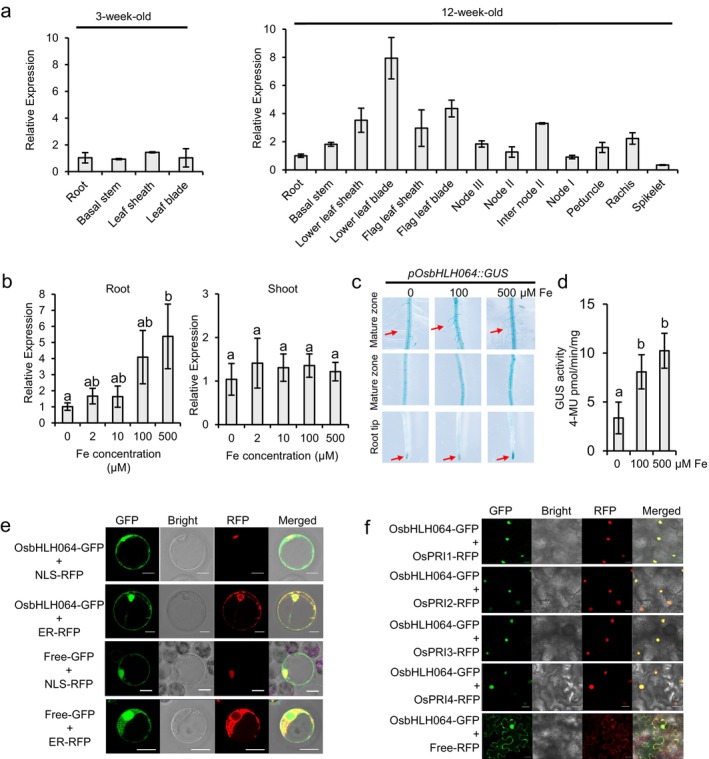
Expression patterns and subcellular localisation of OsbHLH064. (a) Temporal and spatial expression analysis of *OsbHLH064* by RT‐qPCR across rice tissues at different developmental stages. Transcript levels were normalised to *OsACTIN2* and presented as relative to root expression at the seedling stage. Values represent mean ± SD (*n* = 3 biological replicates). (b) *OsbHLH064* expression under varying Fe conditions in roots and shoots. 7‐day‐old rice seedlings were grown in Fe deficiency (0 μM), Fe control (100 μM) or Fe excess (500 μM) nutrient solutions for 7 days. Transcript levels of *OsbHLH064* in roots and shoots were analysed separately by RT‐qPCR. Different letters indicate statistically significant differences between treatments (one‐way ANOVA, followed by Tukey's HSD test, *P* < 0.05). Values are presented as mean ± SD (*n* = 3 biological replicates). (c) Histochemical GUS staining of *pOsbHLH064::GUS* transgenic rice seedlings exposed to Fe deficiency (0 μM), Fe control (100 μM) or Fe excess (500 μM) conditions. GUS activity in roots was modulated by Fe availability, with staining intensity reflecting changes in *OsbHLH064* promoter activity. Red arrows indicate differential GUS staining in root hair and root tip regions under different Fe conditions. (d) Quantitative GUS activity assay *of pOsbHLH064::GUS* roots under different Fe conditions. (e) Subcellular localisation of OsbHLH064‐GFP fusion protein in rice protoplasts. GFP signals co‐localised with both the ER and nuclear marker (NLS‐RFP), indicating dual localisation to the nucleus and ER. Scale bars = 20 μm. (f) IVc subgroup bHLHs promote the nuclear localisation of OsbHLH064. OsbHLH064‐GFP was co‐transformed with Free‐RFP, OsPRI1‐RFP, OsPRI2‐RFP, OsPRI3‐RFP, or OsPRI4‐RFP into tobacco leaves. Scale bars = 20 μm.

To investigate the expression pattern of *OsbHLH064*, RT‐qPCR was performed in various rice tissues at different developmental stages. As shown in Figure 2a, *OsbHLH064* was expressed in all tested tissues from 3‐ and 12‐week‐old plants. To assess whether *OsbHLH064* expression is responsive to Fe availability, 7‐day‐old rice seedlings were transferred to ½‐strength Kimura B (½ KB) medium containing different concentrations of Fe (0, 2, 10, 100 and 500 μM) for 7 days. Roots and shoots were harvested separately for expression analysis. As shown in Figure 2b, the expression of *OsbHLH064* in the shoots remained largely unchanged in response to Fe supply. In contrast, its expression in roots was induced under Fe excess conditions, but downregulated under Fe deficiency. To further characterise the spatiotemporal expression of *OsbHLH064*, a promoter–GUS fusion construct (*pOsbHLH064:GUS*) was introduced into WT plants. Histochemical GUS staining revealed that *OsbHLH064* is broadly expressed in roots (Figure 2c). Moreover, GUS staining intensity was enhanced under Fe‐sufficient conditions and reduced under Fe‐deficiency conditions(Figure 2c), corroborating the RT‐qPCR results. Consistently, quantitative GUS activity assays showed a similar trend, with significantly higher enzymatic activity under Fe‐sufficient or Fe‐excess conditions and markedly reduced activity under Fe deficiency, further confirming that OsbHLH064 expression is tightly regulated by Fe availability (Figure 2d).

### 2.3 OsPRIs Facilitate the Nuclear Localisation of OsbHLH064

The subcellular localisation of a given transcription factor is one of the key features that shape its regulatory function. OsbHLH064 subcellular localisation analysis was performed using the OsbHLH064‐GFP fusion construct transiently expressed in rice protoplasts. The results showed that OsbHLH064 localises to the nucleus and endoplasmic reticulum (Figure 2e). Interestingly, previous studies showed that OsPRIs are exclusively localised in the nucleus (Zhang et al. 2017; Li et al. 2025). Given that BiFC assays demonstrated their interaction occurs specifically in the nucleus (Figure 1b), we hypothesised that OsPRIs may influence the nuclear localisation of OsbHLH064. To test this hypothesis, transient co‐expression in *N. benthamiana* leaves was performed. Co‐expression of free *RFP* with *OsbHLH064‐GFP* resulted in OsbHLH064 distribution in both the nucleus and the cytoplasmic region (Figure 2f), whereas co‐expression of IVc bHLHs fused to RFP with *OsbHLH064‐GFP* led to predominant accumulation of OsbHLH064 in the nucleus. Together, these results indicate that IVc subgroup bHLHs could promote the nuclear accumulation of OsbHLH064, potentially enhancing its transcriptional regulatory function.

### 2.4 Loss of Function of *OsbHLH064* Alters Basal Expression of Iron Homeostasis‐Related Genes

To investigate the biological roles of *OsbHLH064* in regulating Fe homeostasis in rice, the *OsbHLH064* gene was edited using the CRISPR/Cas9 system and two independent mutant lines (i.e., *osbhlh064‐1* and *osbhlh064‐2*) were identified through sequencing analysis. The *osbhlh064‐1* mutant contained a 1‐bp deletion, while *osbhlh064‐2* harboured a 1‐bp insertion at the second exon, both leading to a premature stop codon and frameshift mutation prior to the conserved bHLH domain (Figures S2a, S3). However, phenotypic analysis revealed no obvious differences between *osbhlh064* mutants and WT under either Fe‐sufficient or Fe‐deficient conditions (Figure S2). Consistently, inductively coupled plasma mass spectrometry (ICP‐MS) measurements showed no significant differences in Fe content between WT and *osbhlh064* mutant roots, young leaves and old leaves grown under both Fe conditions (Figure S2d–f). Furthermore, analysis of mature brown rice grains showed no significant difference in Fe content between WT and mutant lines (Figure S4). These results suggest that OsbHLH064 function might be related to the modulation of the repartition of inter‐ and/or intracellular Fe pools and/or might be masked by functional redundancy with other IVb bHLH family members.

**FIGURE 3:**
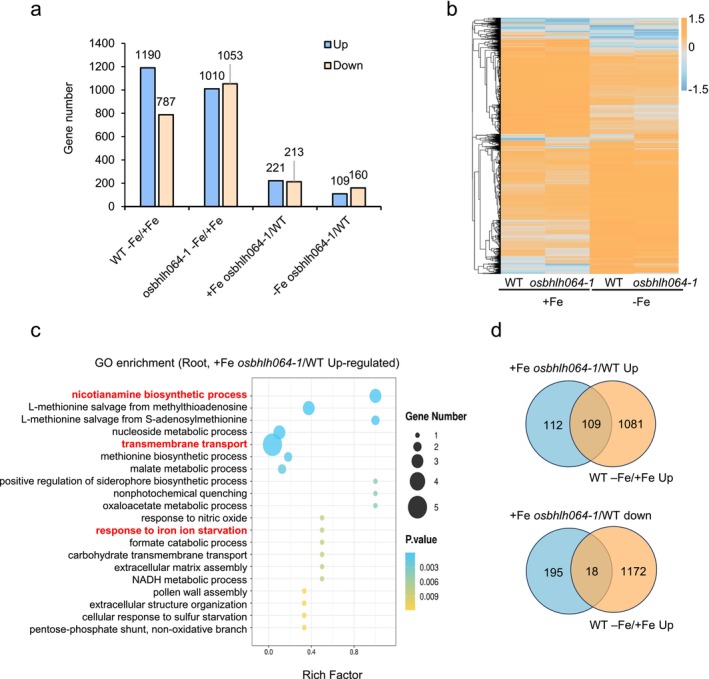
Transcriptomic analysis reveals altered expression of iron homeostasis genes in the *osbhlh064‐1* mutant. (a) Number of differentially expressed genes (DEGs) identified in wild type (WT) and *osbhlh064‐1* grown under Fe‐sufficient (100 μM) and Fe‐deficient (0 μM) conditions. (b) Heatmap showing the expression patterns of DEGs in roots of WT and *osbhlh064‐1* grown under both Fe‐sufficient (100 μM) and Fe‐deficient (0 μM) conditions. Gene expression levels are displayed as Z‐scores of log_2_‐transformed FPKM values. (c) Gene Ontology (GO) enrichment analysis of genes upregulated in roots of *osbhlh064‐1* compared to WT grown under Fe‐sufficient conditions. (d) Overlap between Fe deficiency‐induced genes in WT roots and DEGs between *osbhlh064‐1* and WT roots under Fe‐sufficient conditions.

We therefore hypothesised that OsbHLH064 activity might lead to subtle changes at the transcriptional level that do not immediately result in visible phenotypes, similar to what has been observed for other IVb bHLH transcription factors (Wang et al. 2025; Li et al. 2025). Hence, to gain deeper insight into the molecular function of *OsbHLH064*, root transcriptome profiling was conducted using RNA‐seq. Compared with WT, 434 DEGs were identified in the *osbhlh064‐1* mutant when grown under Fe‐sufficient conditions (221 upregulated and 213 downregulated) and 269 DEGs when grown under Fe‐deficient conditions (109 upregulated, and 160 downregulated) (Figure 3a,b, Table S1). GO enrichment analysis was conducted for these four DEG sets and, among them, only the upregulated genes under Fe‐sufficient conditions showed significant enrichment of biological processes directly related to Fe homeostasis, including ‘nicotianamine biosynthetic process,’ ‘transmembrane transport,’ and ‘response to iron ion starvation’ (Figures 3c and S5, Table S2). This later observation suggests a potential role for OsbHLH064 in regulating plant Fe homeostasis when grown under Fe replete condition.

To further dissect the role of OsbHLH064 in the regulation of Fe homeostasis, we used the Fe deficiency induced gene set from WT roots as a reference and systematically compared it with DEGs identified in the root of *osbhlh064‐1* mutant relative to the WT under both Fe‐sufficient and Fe‐deficient conditions (Figure 3d). Under Fe‐sufficient conditions, 109 of the 221 upregulated genes in the mutant (Figure 3a) overlapped with Fe deficiency‐induced genes from WT plants. This gene set includes numerous key components of the Strategy II Fe uptake pathway, such as *OsYSL15*, *OsENA1* and *OsTOM1*, as well as genes involved in the biosynthesis of NA and DMA, including *OsNAS1*, *OsNAS2*, *OsNAS3*, *OsNAAT1* and *OsDMAS1* (Table 1). Additionally, the expression of several genes encoding enzymes involved in the Yang cycle, which provides SAM for NA and DMA biosynthesis, was also upregulated. The expression of genes involved in long‐distance Fe translocation, such as *OsNRAMP1* and *OsYSL2*, was also significantly induced (Table 1). In addition to these genes, the expression of several known transcriptional regulators of Fe homeostasis (i.e., *OsIRO2*, *OsIRO3*, *OsFIT*, *OsbHLH061*, *OsbHLH062* and *OsbHLH133*) and Fe signalling components (i.e., *OsHRZ1*, *OsHRZ2*, *OsIMA1* and *OsOPT7*) was upregulated in *osbhlh064‐1* mutant under Fe sufficient conditions (Table 1). Under Fe‐deficient conditions, the expression levels of the above‐described Fe homeostasis related genes were comparable between the WT and the *osbhlh064‐1* mutant (Table 1). This indicates that the loss of *OsbHLH064* function does not impair the transcriptional induction of the above‐described Fe‐homeostasis related genes in response to Fe deficiency. Instead, it shows that OsbHLH064 function primarily affects the basal expression of Strategy II associated Fe acquisition, translocation and signalling genes under Fe‐sufficient conditions in roots. RT‐qPCR analysis of representative genes confirmed RNA‐seq results (Figure S6). These findings underscore the role of *OsbHLH064* as a regulator of Fe homeostasis under Fe replete growth conditions.

**TABLE 1:** Expression patterns of known Fe homeostasis‐related genes in the roots of WT and *osbhlh064‐1* mutant plants. Green indicates significantly upregulated genes, while blue indicates significantly downregulated genes.

Locus	Annotation	+Fe			‐Fe		
		WT	osbhlh064‐1	log_2_FC	WT	osbhlh064‐1	log_2_FC
Strategy I Fe acquisition							
LOC_Os03g46470	*OsIRT1*	0.10	0.56	2.46	3.90	4.05	0.00
LOC_Os03g46454	*OsIRT2*	0.23	1.44	2.67	26.64	19.06	–0.52
LOC_Os03g37490	*OsPEZ1*	12.19	10.77	–0.11	3.65	4.30	0.19
LOC_Os03g37640	*OsPEZ2*	36.60	42.06	0.22	40.09	44.01	0.11
LOC_Os07g15460	*OsNRAMP1*	13.01	100.74	3.02	374.57	421.52	0.18
Strategy II Fe acquisition							
LOC_Os02g43410	*OsYSL15*	5.75	66.51	3.45	278.47	301.73	0.11
LOC_Os04g45900	*OsYSL16*	11.41	15.60	0.52	28.85	24.25	–0.28
LOC_Os11g05390	*OsENA1*	13.57	51.65	1.99	115.35	120.91	0.04
LOC_Os06g48060	*OsENA2*	26.45	30.95	0.29	56.75	52.48	–0.15
LOC_Os11g04020	*OsTOM1*	95.16	637.62	2.80	1146.64	1489.94	0.35
Yang cycle							
LOC_Os06g02220	*OsMTN*	33.32	90.49	1.51	213.54	266.63	0.28
LOC_Os12g39860	*OsAPT1*	168.42	472.15	1.55	987.69	1158.22	0.19
LOC_Os04g57400	*OsMTK1*	33.70	108.08	1.74	312.23	343.12	0.10
LOC_Os04g57410	*OsMTK2*	10.05	39.39	2.03	105.10	111.06	0.04
LOC_Os11g11050	*OsIDI2*	16.93	61.80	1.93	174.72	246.87	0.46
LOC_Os11g29370	*OsDEP*	84.70	475.16	2.55	1308.61	1566.19	0.22
LOC_Os03g06620	*OsIDI1*	54.07	134.22	1.38	386.68	446.31	0.17
LOC_Os06g29180	*OsFDH*	98.94	294.64	1.64	811.06	912.06	0.13
LOC_Os09g28050	*OsIDI4*	38.68	108.49	1.91	547.59	658.63	0.20
LOC_Os05g04510	*OsSAM1*	682.35	804.54	0.30	712.42	737.99	0.01
LOC_Os01g22010	*OsSAM2*	244.33	402.11	1.17	1118.78	1077.18	0.07
LOC_Os12g38270	*OsIDS1*	95.74	709.20	2.96	1689.54	1707.47	–0.02
LOC_Os04g24140	*OsRPI*	38.47	133.23	1.85	297.56	329.56	0.10
DMA synthesis							
LOC_Os03g19427	*OsNAS1*	89.72	1018.17	3.56	2878.40	3126.50	0.08
LOC_Os03g19420	*OsNAS2*	111.11	1296.32	3.60	3844.45	4179.81	0.08
LOC_Os07g48980	*OsNAS3*	8.88	25.02	1.56	56.16	66.53	0.21
LOC_Os02g20360	*OsNAAT1*	23.97	277.70	2.67	829.69	938.62	0.18
LOC_Os03g13390	*OsDMAS1*	33.41	163.12	2.35	530.36	561.95	0.05
Iron translocation							
LOC_Os02g43370	*OsYSL2*	14.43	92.84	2.63	401.71	446.23	0.10
LOC_Os07g15460	*OsNRAMP1*	13.01	100.74	3.02	374.57	421.52	0.18
Iron storage							
LOC_Os11g01530	*OsFER1*	7.87	4.22	–1.03	1.45	1.58	0.10
LOC_Os12g01530	*OsFER2*	7.07	3.89	–0.80	1.22	1.54	0.30
LOC_Os04g38940	*OsVIT1*	3.22	2.39	–0.35	2.93	3.28	–0.03
LOC_Os09g23300	*OsVIT2*	7.33	5.50	–0.35	5.58	13.52	1.23
Transcriptional factor							
LOC_Os07g35870	*OsPRI4*	31.12	30.01	0.07	28.62	31.01	0.12
LOC_Os05g38140	*OsPRI2*	124.81	103.14	–0.21	61.13	53.77	–0.22
LOC_Os02g02480	*OsPRI3*	28.38	26.13	–0.03	14.96	15.90	0.09
LOC_Os08g04390	*OsPRI1*	87.16	83.83	0.01	77.19	84.22	0.09
LOC_Os11g38870	*OsbHLH061*	18.93	8.87	–0.61	2.92	2.84	–0.19
LOC_Os07g43530	*OsbHLH062*	9.09	11.87	0.66	13.47	17.06	0.29
LOC_Os03g26210	*OsIRO3*	18.19	43.94	1.34	134.16	141.43	–0.02
LOC_Os02g23823	*OsbHLH064*	22.56	18.55	–0.41	8.96	8.30	–0.16
LOC_Os01g72370	*OsIRO2*	13.36	77.74	2.60	387.26	389.91	–0.14
LOC_Os04g31290	*OsFIT*	7.45	27.14	1.93	59.21	56.47	–0.11
LOC_Os12g32400	*OsbHLH133*	1.02	5.44	2.44	61.24	57.74	–0.13
LOC_Os08g01090	*OsIDEF1*	14.99	10.30	–0.47	13.30	15.42	0.25
LOC_Os05g35170	*OsIDEF2*	313.59	316.94	0.09	283.30	299.96	0.05
Other genes							
LOC_Os01g49470	*OsHRZ1*	13.78	29.67	0.89	72.53	78.63	0.11
LOC_Os05g47780	*OsHRZ2*	26.63	36.46	0.52	157.04	187.66	0.22
LOC_Os12g18410	*OsMIR*	1.97	25.03	4.09	411.54	386.21	–0.13
LOC_Os01g45914	*OsIMA1*	3.37	7.59	1.23	259.89	239.89	–0.15
LOC_Os03g54000	*OsOPT7*	9.15	36.62	1.85	201.18	166.66	–0.32

To investigate potential functional redundancies between OsbHLH064 and other IVb bHLHs, we compared the transcriptomic profiles of the *osbhlh064*‐1 mutant with a previously reported *osbhlh061* mutant under Fe‐sufficient conditions (Wang, Ye, et al. 2022). A total of 41 genes were commonly upregulated in both mutants. It included key components of the Strategy II Fe acquisition system (e.g., *OsYSL15*, *OsNRAMP1*, *OsTOM1*, *OsENA1*, *OsNAS1*, *OsNAS2*, *OsNAAT1* and *OsDMAS1*), as well as transcriptional regulators, including *OsIRO2*, *OsIRO3* and *OsIMA1* (Figure S7a, Table S1). The consistent upregulation of these genes suggests that the two IVb bHLH transcription factors may redundantly repress a common set of target genes to prevent Fe overaccumulation under sufficient conditions.

### 2.5 Overexpression of *OsbHLH064* Results in Hypersensitivity to Iron Deficiency

**FIGURE 4:**
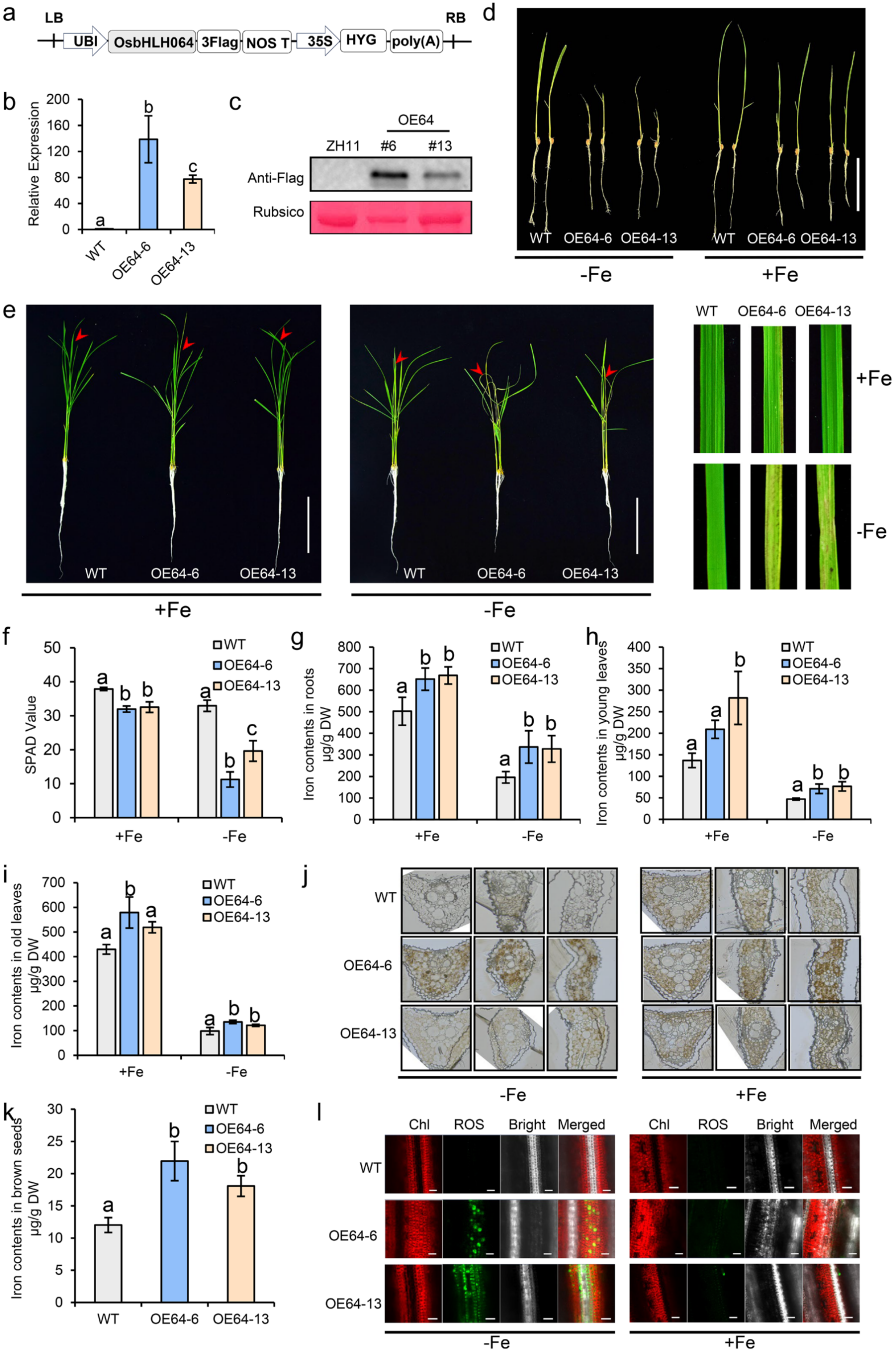
Phenotypic analysis of *OsbHLH064* overexpression lines. (a) Schematic representation of the *OsbHLH064* overexpression construct used for rice transformation. The *OsbHLH064* coding sequence was fused with a C‐terminal 3 × Flag tag and driven by the maize ubiquitin promoter. (b) Transcript levels of *OsbHLH064* in two independent overexpression lines (OE64‐6 and OE64‐13) determined by RT‐qPCR. (c) Immunoblot analysis of OsbHLH064‐Flag protein accumulation in OE lines using anti‐flag antibody; Rubisco band (from Ponceau S staining) serves as the loading control. (d) Phenotypes of 7‐day‐old wild‐type (WT) and OsbHLH064‐overexpressing (OE) seedlings grown under Fe‐deficient (–Fe, 0 μM) and control (+Fe, 100 μM) conditions. Scale bars = 10 cm. (e) Phenotypic comparison of 3‐week‐old WT and OE seedlings grown under Fe‐deficient (–Fe, 0 μM) and control (+Fe, 100 μM) conditions for 3 days. Scale bars = 10 cm. (f) Quantification of SPAD values in WT and OE lines under Fe‐deficient and Fe‐sufficient conditions. (g) Iron concentrations in roots of WT and OE lines (h) Iron contents in old leaves of WT and OE lines. (i) Iron contents in young leaves of WT and OE lines. (j) Histochemical detection of Fe accumulation in leaf cross‐sections of WT and OE plants using Perls’ Prussian blue staining enhanced with DAB. (k) Iron concentrations in brown rice of WT and OE lines grown in the field (paddy conditions, 2024). (l) Reactive oxygen species (ROS) in leaf tissues were visualised using the fluorescent probe H_2_DCFDA. Green fluorescence indicates ROS accumulation, while red fluorescence represents chlorophyll autofluorescence. Scale bars = 20 μm. In B, F, H and I, values are presented as mean ± SD (*n* = 3 biological replicates). Different letters in each group indicate significant differences (*P* < 0.05, one‐way ANOVA, followed by Tukey's HSD test, *P* < 0.05).

To investigate the potential physiological consequences of *OsbHLH064* overexpression, we generated transgenic rice lines constitutively expressing *OsbHLH064* under the control of the ubiquitin promoter (Figure 4a). Two independent lines (i.e., OE64‐6 and OE64‐13) with moderate expression levels were selected for subsequent analysis, as lines with higher expression exhibited lethality and failed to set seed (Figures 4b,c and S8).

When 3 day old seedlings were subjected to Fe‐deficient conditions for 4 days, the overexpression lines rapidly developed severe chlorosis and wilting, with most seedlings dying shortly thereafter. Even under Fe‐sufficient conditions, the overexpression seedlings appeared slightly smaller than WT plants (Figure 4d). To further assess the effects of overexpression at later developmental stages, 3 week old seedlings were exposed to Fe‐sufficient or Fe‐deficient conditions for 5 days (Figure 4e). Under Fe‐sufficient conditions, young leaves of *OsbHLH064* overexpressing lines displayed lower SPAD value than those of the WT under Fe‐sufficient conditions (Figure 4f). When subjected to Fe‐deficiency conditions, they exhibited early onset of chlorosis (i.e., within 1 to 2 days), followed by the appearance of dark brown lesion. The lesions gradually spread toward the leaf base, and by day 5, showed tissue collapse and necrosis, leading to partial or complete death of the young leaves. Since these symptoms appeared more rapidly and severely in OE64‐6 line than in OE64‐13 line, it is likely that a positive correlation exists between the expression level of *OsbHLH064* and the hypersensitivity to Fe‐deficiency (Figure 4e). In contrast, WT plants showed only mild chlorosis under the same conditions, without lesion formation or tissue death (Figure 4e). Taken together, these data revealed that *OsbHLH064* overexpression confers hypersensitivity to Fe deficiency.

### 2.6 Overexpression of *OsbHLH064* Triggers Iron Overload and Reactive Oxygen Species Burst Under Iron Deficiency Conditions

Given the rapid onset of leaf chlorosis and necrotic lesions in *OsbHLH064* overexpression lines under Fe deficiency, we hypothesised that excessive ROS might underlie the observed cell death phenotype. NBT and DAB staining revealed pronounced accumulation of superoxide (O_2_^−^) and hydrogen peroxide (H_2_O_2_), respectively, in the overexpression lines compared to the WT, with markedly stronger signals under Fe‐deficient conditions (Figure S9). Trypan blue staining further showed increased cell death in leaves of *OsbHLH064* overexpressing lines, consistent with ROS‐induced membrane damage (Figure S9). To confirm ROS overaccumulation at the cellular level, we employed H_2_DCFDA fluorescent staining. As expected, *OsbHLH064* overexpression lines exhibited enhanced green fluorescence compared to WT plants, indicating elevated ROS levels (Figure 4l). The fluorescence signal was markedly stronger under Fe‐deficient conditions than under Fe‐sufficient conditions, suggesting that Fe deficiency further amplifies ROS accumulation in the overexpression lines. These results support that *OsbHLH064* overexpression induces ROS overproduction and oxidative stress, which likely underlies the hypersensitive phenotype observed under Fe‐deficient conditions. To assess whether this ROS‐induced damage was associated with ferroptosis‐like cell death, we measured the glutathione (GSH) content in leaves. Surprisingly, GSH level was increased in the overexpression lines under Fe‐deficient conditions compared to the WT (Figure S10), suggesting that cell death in overexpressing plants is unlikely to be mediated by GSH depletion and thus not directly linked to ferroptosis.

To determine whether the hypersensitive phenotype was due to altered Fe accumulation, we measured Fe content in both shoots and roots of WT and *OsbHLH064* overexpression lines after 5 days of different Fe concentration treatment. The results showed that *OsbHLH064* overexpression significantly increased Fe accumulation in roots under both Fe‐sufficient and Fe‐deficient conditions (Figure 4g). Consistently, Fe concentrations in shoots, including both old and young leaves, were also markedly higher in overexpression lines compared to the WT under both Fe treatment conditions (Figure 4h,i). Furthermore, to investigate whether intracellular Fe localisation was affected, we performed histochemical staining of leaf cross‐sections using Perls' Prussian blue enhanced with DAB. The staining confirmed the accumulation results, showing visibly stronger signals in overexpression lines than in WT under both Fe‐sufficient and Fe‐deficient conditions (Figure 4j). Under Fe‐deficient conditions, Fe deposition appeared more pronounced around chloroplast regions in the overexpression lines, suggesting potential alterations in local Fe distribution(Figure 4j). Interestingly, despite the higher Fe accumulation under Fe sufficient conditions than under Fe deficiency, no severe damage was observed, suggesting that increased Fe levels alone were not sufficient to trigger extensive oxidative damage in *OsbHLH064* overexpressing lines.

To investigate whether the altered Fe accumulation extends to reproductive tissues, we measured Fe content in brown rice grains from WT and *OsbHLH064* overexpression lines. Remarkably, overexpression of *OsbHLH064* led to a significant increase in Fe concentration in grains compared to WT plants (Figure 4k, Figure S11), indicating that OsbHLH064 not only modulates Fe homeostasis in vegetative tissues but also contributes to enhanced Fe allocation to seeds. In addition to Fe, the concentrations of Zn and Mn were also elevated in grains of the overexpression lines, whereas Cu and Cd levels remained unchanged (Figure S11). Importantly, despite the elevated Fe levels in grains, no negative impact on major agronomic traits was observed (Figure S11). Field evaluations conducted under standard paddy conditions showed that key yield‐related parameters, including seed‐setting rate and per‐plant grain yield, were comparable between overexpression lines and WT (Figure S11). These results demonstrate that the gain in Fe accumulation does not come at the cost of growth or productivity. Together, these findings suggest that OsbHLH064 is a promising genetic target for iron biofortification in rice, enabling enhanced Fe allocation to grains without compromising agronomic performance.

### 2.7 Overexpression of *OsbHLH064* Disrupts the Transcriptional Regulation of Iron Homeostasis

**FIGURE 5:**
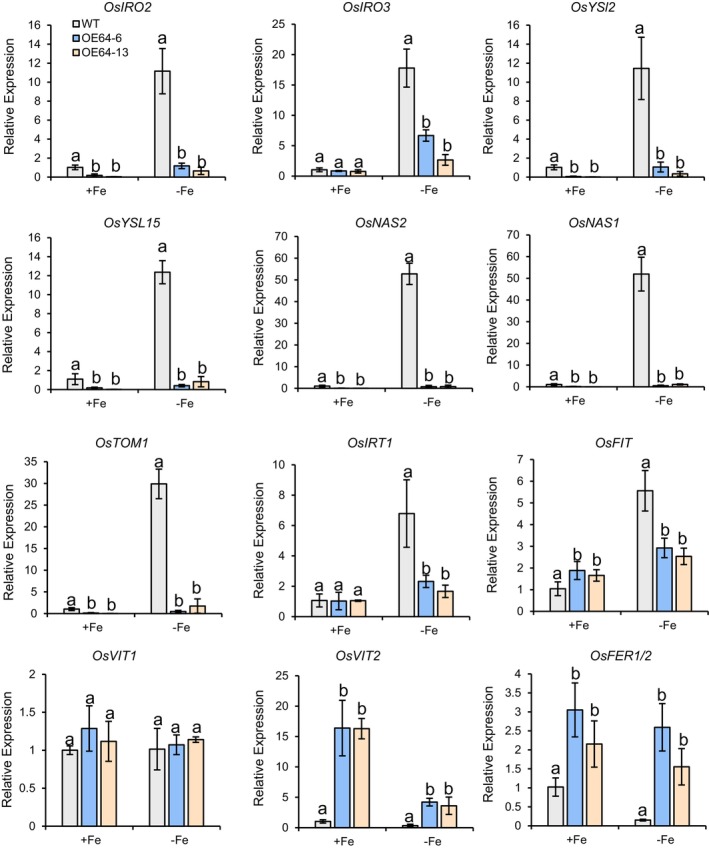
Expression analysis of Fe homeostasis‐related genes in OsbHLH064 overexpression lines. Relative transcript levels of 3 genes encoding regulatory proteins (i.e., *OsIRO2*, *OsIRO3* and *OsFIT* transcription factors) and 6 encoding structural proteins involved in the maintenance of Fe homeostasis (i.e., *OsYSL15*, *OsYSL2*, *OsNAS1*, *OsNAS2*, *OsTOM1* and *OsIRT1*) in the roots of 3‐week‐old WT and *osbhlh064* mutant seedlings grown under control (+Fe, 100 μM) and Fe‐deficiency (–Fe, 0 μM) conditions for 3 days. Expression levels were determined by RT‐qPCR and normalised to *OsACTIN2*. Values are means ± SD (*n* = 3 biological replicates). Different letters indicate statistically significant differences between genotypes under the same treatment (*P* < 0.05, one‐way ANOVA, followed by Tukey's HSD test).

To further elucidate the molecular basis underlying the Fe deficiency induced hypersensitivity observed in *OsbHLH064* overexpression lines, the expression of key genes associated with Fe homeostasis were analysed (Figure 5). RT‐qPCR analysis revealed that the expression of genes encoding proteins involved in Fe uptake (Strategy II), including *OsNAS1*, *OsNAS2*, *OsYSL15* and *OsTOM1*, was markedly downregulated in *OsbHLH064* overexpression lines when compared to WT plants under both Fe‐sufficient and Fe‐deficient conditions (Figure 5). Similarly, the expression of the key positive regulator *OsIRO2* was significantly lower in the *OsbHLH064* overexpression lines than in the WT under both Fe‐sufficient and Fe‐deficient conditions (Figure 5). Interestingly, the expression of *OsIRO3*, which encodes a negative regulator of Fe homeostasis, remained comparable between *OsbHLH064* overexpression lines and WT under Fe‐sufficient conditions, but was markedly reduced under Fe‐deficient conditions (Figure 5). *OsIRT1*, which encodes a key Fe transporter involved in Fe uptake (Strategy I), showed no significant change in transcript levels under Fe‐sufficient conditions in the *OsbHLH064* overexpression lines (Figure 5). However, under Fe‐deficient conditions, *OsIRT1* expression was markedly repressed in the overexpression lines, suggesting a disrupted response to Fe deficiency. *OsFIT*, which encodes a central transcriptional regulator of Fe uptake, exhibited a contrasted expression pattern. For instance, *OsFIT* expression was higher in *OsbHLH064* overexpressing lines than in the WT under Fe sufficiency and lower under Fe deficiency condition (Figure 5). It is noteworthy that the induction of the expression of the above‐described genes in response to Fe deficiency was compromised in the *OsbHLH064* lines. Since we have observed that *OsbHLH064* overexpression lines accumulates more Fe than the WT under Fe deficiency condition, we examined the expression of genes involved in Fe storage (i.e., *OsFER1/OsFER2*) and sequestration (i.e., *OsVIT1* and *OsVIT2*). *OsFER1/OsFER2* was induced in *OsbHLH064* overexpression lines regardless of Fe availability (Figure 5). The expression of *OsVIT2* followed the same expression pattern while *OsVIT1* expression remained comparable between overexpression lines and the WT in both Fe regimes (Figure 5). In addition, Fe homeostasis related genes such as *OsIMA1*, *OsMIR*, *OsOPT7* and *OsNRAMP1* were downregulated in the overexpression lines under Fe deficiency conditions (Figure S12).

### 2.8 Genome‐Wide Identification of OsbHLH064 Binding Sites

**FIGURE 6:**
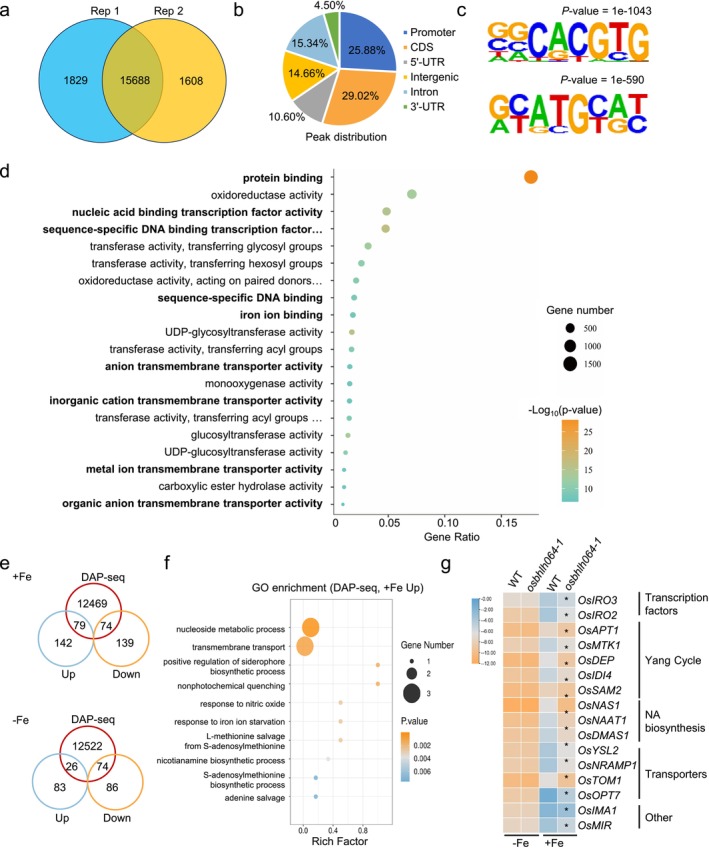
Genome‐wide identification of OsbHLH064‐binding targets by DAP‐seq. (a) Venn diagram showing overlapping peaks identified in two independent DAP‐seq replicates for OsbHLH064. High‐confidence binding peaks were defined by the intersection of the two datasets. (b) Genomic distribution of OsbHLH064‐binding peaks relative to gene features, including promoter, coding sequence (CDS), 5′‐end untranslated region (5′‐UTR), intron, 3′‐end untranslated region (3′‐UTR) and intergenic regions. (c) Sequence logo of the enriched DNA‐binding motif identified from OsbHLH064‐bound in promoter and 5′‐UTR regions using MEME‐ChIP. The *G‐box* (i.e., *CACGTG*) motif is significantly enriched. (d) Gene Ontology (GO) enrichment analysis of OsbHLH064 target genes. The top enriched GO terms are displayed based on ‐log_10_(*P* value). (e) Venn diagram showing overlap between DAP‐seq targets and genes differentially expressed in the mutant under Fe‐sufficient or Fe‐deficient conditions. (f) GO term enrichment analysis of the overlapping genes from DAP‐seq and up‐regulated genes in *osbhlh064‐1* mutant grown under Fe‐sufficient conditions. (g) Heatmap showing the expression patterns of known Fe homeostasis‐related genes identified as OsbHLH064‐binding targets. NA: Nicotianamine. * indicates a statistically significant difference.

Given that the expression of numerous Fe homeostasis‐related genes was misregulated in both *OsbHLH064* knockout and overexpression lines, we sought to identify the direct downstream targets of OsbHLH064 at the genome‐wide level. To this end, we performed DNA affinity purification sequencing (DAP‐seq), a powerful technique that enables the identification of transcription factor binding sites across the genome within their native DNA context (Bartlett et al. 2017). Two independent biological replicates, using genomic DNA from roots of plants grown under Fe‐sufficient condition, consistently identified 15 688 enriched binding peaks, corresponding to 12 622 putative target genes (Figure 6a, Table S3). Analysis of OsbHLH064 binding sites revealed that while a portion were located in 5′‐UTRs (10.6%), 3′‐UTRs (4.5%), exons (29.0%), introns (15.3%) and intergenic regions (14.7%), a considerable number (25.9%) were enriched in promoter regions (up to 2 kb upstream from the transcription start site) (Figure 6b), consistent with its role as a transcription factor involved in the regulation of gene expression. Motif enrichment analysis at the promoter and 5′‐UTR regions revealed that the most significantly enriched binding motif among OsbHLH064 DAP‐seq peaks was *CACGTG* (Figure 6c), a canonical *G‐box* element known to be specifically recognised by bHLH transcription factors. This result supports the conclusion that OsbHLH064 binds directly to *G‐box*‐containing promoter regions to regulate target gene expression. Gene Ontology (GO) enrichment analysis of OsbHLH064 target genes revealed significant enrichment in ion transport and homeostasis related categories, including terms such as ‘iron ion binding’, ‘metal ion transmembrane transporter activity’, ‘anion transmembrane transporter activity’ and ‘inorganic cation transmembrane transporter activity’ (Figure 6d, Table S4), indicating a key role for OsbHLH064 in regulating Fe transport.

We then searched for genes that were both bound by OsbHLH064 (DAP‐seq) and differentially expressed in *osbhlh064* mutants, in both Fe‐sufficient and Fe‐deficient conditions (RNA‐seq). The aim being to identify high‐confidence direct targets of OsbHLH064 involved in the regulation of Fe homeostasis. This analysis revealed 79 and 74 genes bound by OsbHLH064 that had their expression induced or repressed in *osbhlh064* mutant plants when grown under Fe‐sufficient conditions, respectively (Figure 6e, Table S5). When grown under Fe‐deficiency, there are 26 and 74 genes that had their expression induced or repressed in mutant plants, respectively (Figure 6e, Table S5). Interestingly, the set of genes that was both upregulated in the *osbhlh064* mutant when grown under Fe‐sufficient conditions and bound by OsbHLH064 was significantly enriched in GO terms related to Fe homeostasis (e.g., ‘transmembrane transport’, “‘response to iron ion starvation’ ‘nicotianamine biosynthetic process’) (Figure 6f). This observation supports the idea that OsbHLH064 is involved in the direct transcriptional regulation of genes involved in Fe uptake, Fe distribution and Fe utilisation, particularly under Fe‐sufficient conditions.

### 2.9 OsbHLH064 Functions as a Direct Transcriptional Regulator of Genes Involved in the Maintenance of Iron Homeostasis

**FIGURE 7:**
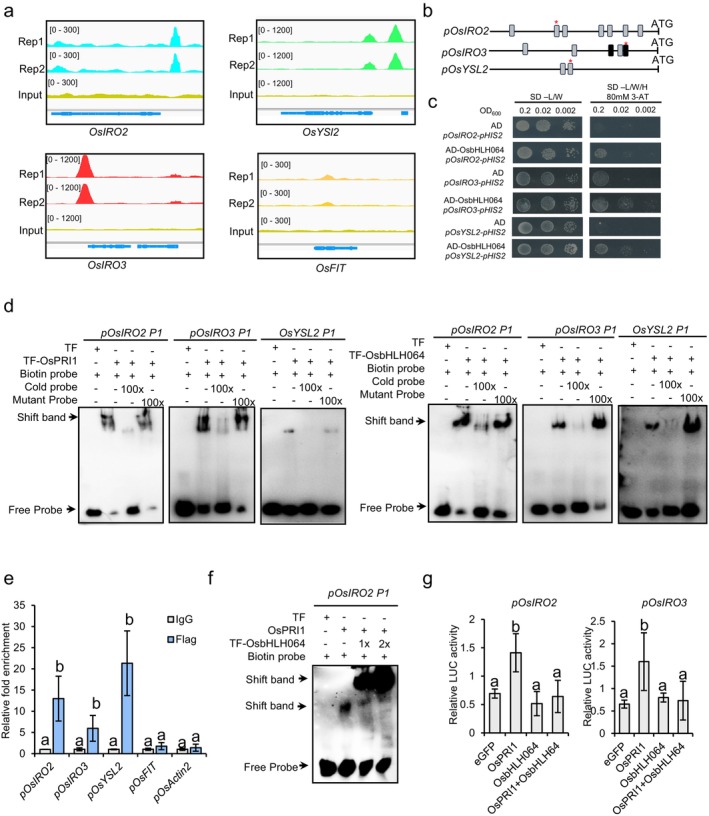
OsbHLH064 directly binds to and represses the transcription of Fe homeostasis‐related genes.(a) DAP‐seq binding profiles showing OsbHLH064 enrichment at the promoters of *OsIRO2*, *OsIRO3* and *OsYSL2*, but not *OsFIT*. (b) Schematic representation of the promoter of *OsIRO2*, *OsIRO3* and *OsYSL2*. Putative *G‐box* and *E‐box* motifs are represented by black and grey rectangles, respectively. (c) Yeast one‐hybrid assay demonstrating the binding of OsbHLH064 to the promoters of *OsIRO2*, *OsIRO3* and *OsYSL2*. Promoter fragments containing putative *E‐box* or G‐box motifs, indicated by red stars in (b), were cloned upstream of the *HIS3* reporter gene. The AD‐OsbHLH064 fusion protein and reporter construct were co‐transformed into Y187 yeast cells. Yeast were grown on SD/−Leu/−Trp/‐His medium supplemented with 3‐amino‐1,2,4‐triazole (3‐AT) to suppress background growth. Yeast growth indicates a specific protein‐DNA interaction. Empty vector pGADT7 was used as a negative control. (d) Electrophoretic mobility shift assays. The biotin‐labelled DNA probe was incubated with the recombinant trigger factor (TF)‐OsbHLH064/OsPRI1 protein. An excess of unlabelled cold‐probe or unlabelled mutated probe was added to compete with the labelled probe. The biotin‐probe incubated with trigger factor protein served as the negative control. (r) ChIP–qPCR analysis showing in vivo enrichment of OsbHLH064 at *OsIRO2*, *OsIRO3* and *OsYSL2* promoter regions in transgenic rice expressing the *pUbi::OsbHLH064‐Flag* construct. Enrichment values are shown relative to the no‐antibody IgG control. (f) EMSA‐based competition assays showing that OsbHLH064 competitively interferes with OsPRI1 binding to the same cis‐regulatory elements. Increasing amounts of TF‐OsbHLH064 protein progressively reduced OsPRI1‐DNA complex formation, indicating direct competition for DNA binding sites. (g) Dual‐luciferase assay *in N. benthamiana* showing that OsbHLH064 represses OsPRI1‐mediated activation of *OsIRO2* and *OsIRO3*. Reporter constructs harbouring the 3 × *OsIRO2* and *OsIRO3 cis*‐element before 35S minimal promoter‐driven LUC genes were co‐infiltrated with effector constructs expressing *OsPRI1* alone or together with *OsbHLH064*. LUC activity was normalised to REN. Values represent means ± SD (*n* = 3 biological replicates). Different letters indicate significant differences (one‐way ANOVA, followed by Tukey's HSD test, *P* < 0.05).

To explore the functional relevance of the high‐confidence OsbHLH064 potential targets identified through the integration of DAP‐seq and transcriptome data, we next focused on individual genes associated with the maintenance of Fe homeostasis in rice. We found among them genes encoding well‐characterised Fe homeostasis regulators such as the transcription factors OsIRO2 and OsIRO3 or the small peptide OsIMA1 (Figures 6g, 7a and S14) (Li, Li, et al. 2022; Ogo et al. 2007; Wang, Ye, et al. 2020; Wang, Itai, et al. 2020). Genes encoding enzymes involved in the methionine (Yang) cycle (i.e., *OsAPT1*, *OsMTK1*, *OsDEP*, *OsIDI4* and *OsSAM2*), the biosynthesis (i.e., *OsNAS1*, *OsNAAT1* and *OsDMAS1*) and transport (i.e., *OsYSL2*) of NA, or involved in Fe uptake (i.e., *OsNRAMP1* and *OsTOM1*) and Fe partitioning (i.e., *OsOPT7* and *OsMIR*), were also found.

To further validate in vivo the interaction between OsbHLH064 and the promoters of its putative target genes, we first performed yeast one‐hybrid (Y1H) assays focusing on *OsIRO2*, *OsIRO3* and *OsYSL2* (Figure 7c). We found that multiple *G‐box* (*CACGTG*) and *E‐box* (*CANNTG*, with *n* = *A*, *C*, *G* or *T*) motifs were distributed across the promoter regions of these genes. We therefore focused on the motifs that were located within the DAP‐seq peak regions (Figure 7a,b). Y1H assays demonstrated that OsbHLH064 could interact in vivo with the promoter fragments of *OsIRO2*, *OsIRO3* and *OsYSL2* (Figure 7c). In vitro experiments (EMSA) confirmed the specific binding of OsbHLH064 to *G‐box* or *E‐box* motifs present in the promoter regions of *OsIRO2*, *OsIRO3* and *OsYSL2* but also in the one of *OsIMA1*, *OsNRAMP1*, *OsOPT7* and *OsMIR* (Figures 7d and S13). ChIP‐qPCR analysis confirmed *in planta* the interaction between OsbHLH064 and the promoter of the above‐described genes (Figures 7e and S14). Together, these results provide strong evidence that OsbHLH064 directly binds to *G‐box* or *E‐box* motifs within the promoters of multiple Fe homeostasis related genes, thereby modulating their transcription.

EMSA experiments revealed that OsbHLH064 and OsPRI1 bind to the same cis‐regulatory sequences present in the promoters of all tested target genes(Figures 7f and S13). Notably, EMSA‐based competition assays further demonstrated that increasing amounts of OsbHLH064 progressively reduced the binding of OsPRI1 to these cis‐elements of the *OsIRO2*, indicating that OsbHLH064 can directly compete with OsPRI1 for the same DNA‐binding sites(Figures 7f and S15). These results suggest a potential competitive and coordinated regulation of downstream gene expression by the two transcription factors. Given that OsbHLH064 also physically interacts with OsPRI1, we hypothesised that OsbHLH064 may modulate the transcriptional activity of OsPRI1. To test this assumption, we performed dual‐luciferase reporter assays using the *cis*‐regulatory sequences derived from *OsIRO2* and *OsIRO3* promoters as representative targets (Figure 7g). Consistent with its role as a positive regulator, OsPRI1 markedly enhanced the transcriptional activity driven by these promoter elements when co‐expressed in *N. benthamiana* leaves. Interestingly, co‐expression of OsbHLH064 significantly suppressed this OsPRI1‐mediated activation, highlighting that OsbHLH064 can effectively inhibit the transactivation capacity of OsPRI1 (Figure 7g). Taken together, these results demonstrate that OsbHLH064 antagonises OsPRI1 transcriptional activity through direct competition for DNA binding and functional interference, thereby fine‐tuning Fe‐responsive gene expression and maintaining iron homeostasis in rice.

## 3 Discussion

The regulation of Fe homeostasis in plants is orchestrated by a complex transcriptional regulatory network involving multiple bHLH transcription factors (Gao and Dubos 2021; Liang 2022). In rice, subgroup IVc bHLHs (i.e., OsPRI1, OsPRI2, OsPRI3 and OsPRI4) function as positive regulators of Fe homeostasis‐related genes, including the *OsIRO2* bHLH transcription factor (Zhang et al. 2017, 2020; Kobayashi et al. 2019). In contrast, three out of four subgroup IVb bHLHs (i.e., OsbHLH061, OsbHLH062 and OsIRO3) have been identified as negative regulators. Herein, we investigated the potential role of the fourth subgroup IVb bHLH member, namely OsbHLH064, in regulating Fe homeostasis.

Loss of function of *OsbHLH064* did not lead to obvious changes in growth performance or Fe accumulation when compared to WT plants (Figure S2). However, transcriptome analysis revealed that many Fe‐deficiency‐responsive genes were significantly upregulated in *osbhlh064* mutants but only when grown under Fe‐sufficient condition (Table 1). These findings suggest that OsbHLH064 is involved in the repression of Fe‐deficiency‐responsive genes expression when Fe is not limiting. Interestingly, similarly to what was observed for *osbhlh064* mutants, *osbhlh061*, *osbhlh062* and *iro3* loss‐of‐function mutants show no obvious phenotypic differences when compared to WT plants when grown under Fe‐sufficient conditions (Carey‐Fung et al. 2022; Wang, Itai, et al. 2020; Liang et al. 2020; Li, Li, et al. 2022; Wang, Ye, et al. 2020; Li et al. 2025; Wang et al. 2025). However, if these mutants display comparable phenotypes, they also exhibit specific features. For instance, unlike what was observed for *osbhlh064*, *osbhlh062* and *iro3*, Fe accumulation is slightly increased in *osbhlh061* shoots when compared to WT plants. In addition, the induction of Fe deficiency responsive genes observed in *osbhlh064*, *osbhlh061* and *iro3* grown in Fe‐replete condition also occurs for *osbhlh062* when grown under Fe deficiency (Carey‐Fung et al. 2022; Wang, Itai, et al. 2020; Liang et al. 2020; Li, Li, et al. 2022; Wang, Ye, et al. 2020; Li et al. 2025; Wang et al. 2025). While characterising *osbhlh062 iro3* double mutants, it was shown that the expression of Fe‐deficiency‐inducible genes and the accumulation of Fe was drastically increased in both roots and shoots, regardless of Fe availability (Li et al. 2025). These later findings suggest that *OsIRO3* and *OsbHLH062* act in a partially redundant and complementary manner to repress the Fe deficiency response and to prevent excessive accumulation of Fe that could be detrimental to the plants (Li et al. 2025). Similarly, transcriptome comparison revealed that *osbhlh064* and *osbhlh061* mutants share a substantial set of commonly upregulated Fe homeostasis‐related genes (Figure S7a, Table S1), and both OsbHLH061 and OsbHLH064 can bind to the promoters of these common target genes(Figures 7 and S7b), supporting that these two IVb bHLHs may function redundantly to regulate the homeostasis of Fe under Fe‐sufficient conditions. Whether or not this is also the case for all the members of this bHLH subgroup will necessitate generating higher‐order mutants between *osbhlh061*, *osbhlh062*, *iro3* and *osbhlh064*.

Overexpression lines provide a complementary approach to uncover potentially redundant functions between IVb bHLHs transcription factors in regulating Fe homeostasis. Overexpression of *OsIRO3*, *OsbHLH061* or *OsbHLH062* leads to the downregulation of Fe deficiency‐inducible genes and increased sensitivity to Fe deficiency, which is consistent with their roles as negative regulators (Wang, Ye, et al. 2022; Wang et al. 2025; Zheng et al. 2010). However, these overexpression lines showed distinct differences in Fe accumulation compared to WT plants. For example, *OsbHLH061* overexpression increased Fe content in roots but decreased it in shoots, whether or not plants were grown under Fe replete or Fe deficiency conditions, indicating that OsbHLH061 negatively regulate root‐to‐shoot translocation of Fe (Wang, Ye, et al. 2022). *OsbHLH062* overexpression also reduces shoot Fe levels without significantly affecting root Fe content, regardless of Fe availability (Wang et al. 2025). Similarly, shoot Fe content is also reduced in *OsIRO3* overexpressing lines, but only when grown under Fe deficiency (Zheng et al. 2010; Li, Li, et al. 2022). Strikingly, the overexpression of *OsbHLH064* showed clear contrast when compared to the overexpression of the other IVb bHLH members. For instance, OsbHLH064 overexpression significantly increase the Fe content in both roots and shoots of plants grown on either Fe sufficient or Fe deficiency conditions (Figure 4). Interestingly, *OsbHLH064* overexpression leads to the downregulation of several genes involved in Strategy II Fe uptake and does not affect the expression of *OsIRT1* (Strategy I), when plants are grown in Fe replete condition(Figure 5). The increased Fe accumulation observed in the overexpression lines may partially derive from Strategy I iron uptake system (e.g., OsIRT1) or other compensatory pathways, which continue to import Fe even when Strategy II genes are repressed. Notably, the accumulated Fe appears to be functionally underutilised, and *OsbHLH064* overexpression lines display atypical Fe‐deficiency symptoms driven by disrupted Fe homeostasis rather than simple Fe scarcity (Figure 5). This observation implies that OsbHLH064 may regulate not only Strategy II‐mediated Fe uptake but also Fe signalling, translocation and utilisation, although the precise molecular mechanisms underlying these effects remain to be elucidated.

*OsbHLH064* overexpression lines also accumulate ROS and develop necrotic lesions on young leaves under Fe‐deficient conditions, a phenotype that is distinct from other IVb bHLH overexpression lines (Figure 4), but similar to that of *iro3* mutants (Wang, Itai, et al. 2020; Carey‐Fung et al. 2022; Li, Li, et al. 2022; Wang, Ye, et al. 2020). Since the buffering capacity for excess Fe is likely maintained and since the total Fe content when grown under Fe‐sufficient conditions is higher than when grown under Fe‐deficient conditions, one can hypothesise that the excess of Fe is not the sole factor triggering the observed ROS burst (Figure 4). Measurements of GSH levels indicated that the observed cell death was not related to ferroptosis (Figure S10) (Distéfano et al. 2022, 2021). Interestingly, similar phenomena were observed in Arabidopsis, where loss of function of the IVc bHLH gene *ILR3* (*IAA‐LEUCINE RESISTANT 3*) or the IVb bHLH gene *PYE* (*POPEYE*) results in a striking leaf bleaching phenotype under Fe‐deficient conditions. This bleaching is caused by excess accumulation of singlet oxygen due to defects in photosystem II (PSII) photoprotection (Akmakjian et al. 2021). Given that OsIRO3 is an ortholog of PYE, it is tempting to speculate that the excessive ROS accumulation and the necrotic lesions observed in *iro3* may also arise from defects in PSII photoprotection when plants are grown under Fe‐deficiency conditions (Li et al. 2025; Wang et al. 2025). Whether or not the necrotic lesions and excessive ROS accumulation occurring in *OsbHLH064* overexpression lines is due to the observed decreased expression of *OsIRO3* remains to be investigated. Additionally, the Fe‐staining analysis indicates that *OsbHLH064* overexpression may disturb subcellular Fe distribution (Figure 4j). In particular, Fe appears to accumulate abnormally around chloroplasts(Figure 4j), a microenvironment with high light flux and active electron transfer, where mislocalised Fe is highly prone to redox cycling. Such perichloroplastal Fe enrichment could therefore amplify localised ROS production. Under Fe‐deficient conditions when chloroplast Fe demand is elevated while photoprotective capacity is weakened, this imbalance would be expected to further destabilise PSII and exacerbate ROS‐mediated damage (Kroh and Pilon 2020). Nevertheless, the precise contribution of mislocalised Fe to ROS accumulation and cellular damage remains to be clarified.

The function of a given transcription factor might also be regulated by its subcellular localisation, a critical mechanism for regulating downstream gene expression in response to environmental fluctuations (Wang et al. 2021; Allen and Strader 2021). For instance, OsIRO3 showed exclusive nuclear localisation, whereas OsbHLH061 and OsbHLH062 were detected in both the nucleus and the cytoplasm (Li, Li, et al. 2022; Li et al. 2025; Wang et al. 2025). Similarly, we found that OsbHLH064 accumulates in both the nucleus and the endoplasmic reticulum (Figure 2e–f). In contrast, rice IVc bHLH proteins (i.e., OsPRI1, OsPRI2, OsPRI3 and OsPRI4) are exclusively localised in the nucleus (Zhang et al. 2020, 2017). OsPRIs can also interact with OsbHLH062 and enhance its nuclear localisation when co‐overexpressed in tobacco leaves (Li et al. 2025). Consistent with these findings, our results showed that OsbHLH064 also physically interacts with IVc bHLH transcription factors, promoting its nuclear localisation (Figure 2f). It is noteworthy that these mechanisms extend to other rice Fe homeostasis related bHLH (i.e., OsFIT and OsIRO2) and are conserved in Arabidopsis (Pu and Liang 2023; Gao et al. 2024; Lei et al. 2020; Li, Lei, et al. 2022). These findings indicate that dynamic nucleocytoplasmic localisation of IVb bHLHs is essential to maintain Fe homeostasis in plants. This conditional nuclear accumulation may provide an efficient and rapid mechanism to fine‐tune Fe homeostasis related gene expression in response to fluctuations in Fe availability, therefore preventing excessive Fe accumulation. Unfortunately, our attempts to examine whether Fe availability affects the subcellular localisation of OsbHLH064, using nuclear–cytoplasmic fractionation in *OsbhLH064* overexpression lines were unsuccessful. Thus, whether these nucleocytoplasmic shifts are directly modulated in vivo by cellular Fe status remains to be further investigated.

OsbHLH064 acts as negative regulator of gene expression. Previous studies have shown that the repressive activity of IVb bHLH transcription factors is associated with the EAR motif located in their C‐terminal region. The EAR motif, is characterised by the consensus sequence patterns LxLxL or DLNxxP, that allows recruiting TOPLESS/TOPLESS‐RELATED co‐repressors, thus leading to the active repression of target genes expression (Kagale and Rozwadowski 2011; Plant et al. 2021). This is for instance the case for OsIRO3 that harbours an EAR motif (i.e., LELKL) allowing the suppression of IVc bHLHs transcriptional activity (Li, Li, et al. 2022). Nonetheless, EAR‐like motifs present in OsbHLH062 (i.e., RELKLF) have been shown to play similar roles (Wang et al. 2025). Similarly, even if OsbHLH061 contains a canonical EAR motif (LRLSLP), this is through its EAR‐like motif (QELQLF) that it interacts with OsTPL/OsTPR co‐repressors to mediate its repressive activity (Wang, Ye, et al. 2022). In this study, we found that OsbHLH064 also contains an EAR‐like motif (i.e., TDLELK) (Figure S16); however, no interaction with OsTPL/OsTPR co‐repressors was observed in yeast (Figure S17). Similarly, despite harbouring a DLNxxP‐type EAR motif, PYE fails to interact with TPL/TPRs. These observations suggest that the mere presence of an EAR‐ or EAR‐like motif is not sufficient to ensure TPL/TPR co‐repressors recruitment and that additional motifs or factors might be required (Pu and Liang 2023). Conversely, this lack of interaction with TPL/TPR co‐repressors might indicate that OsbHLH064 and PYE are passive repressors. Passive repressors function primarily by mechanisms such as steric hindrance, where they block the binding or activity of transcriptional activators at target promoters (Krogan and Long 2009). This is for instance what is proposed for PYE that inhibits the activity of IVc bHLH proteins, possibly through competitive binding to target DNA sequences or most likely by forming inactive complex at their DNA binding sites (Pu and Liang 2023). Since OsbHLH064 lacks intrinsic transcriptional activity in both yeast and plant systems (Figure S18), heterodimerises with IVc bHLH proteins (Figure 1) and binds to *cis*‐regulatory sequences targeted by IVc bHLHs to negatively regulate the expression of downstream target genes (Figure 7d and S13), it is likely that OsbHLH064 functions, like PYE, as a passive transcriptional repressor. Indeed, further investigation will be necessary to determine if, like OsbHLH062 and OsIRO3, OsbHLH064 could repress the expression of its target genes via a TPL‐independent mechanism that relies on the recruitment of the E3 ubiquitin ligase OsHRZ1, thereby promoting OsPRIs degradation (Li et al. 2025). Such passive repression mechanism provides an additional regulatory layer in maintaining Fe homeostasis, highlighting the functional diversification within the IVb bHLH subgroup.

IVc and IVb bHLHs are first considered as part of a regulatory loop aiming at regulating their activity and the one of additional regulatory proteins such as OsFIT and OsIRO2. In this study, we found that OsbHLH064 likely functions as a direct transcriptional repressor of *OsIRO2* and *OsIRO3* (Figure 7). These results are in line with the position of OsbHLH064 within the Fe homeostasis transcriptional regulatory network (Li et al. 2025). Nonetheless, we also found among the direct targets of OsbHLH064 several structural genes involved in the maintenance of Fe homeostasis (e.g., *OsYSL2*, *OsMIR*, *OsNRAMP1* and *OsOPT7*) (Figures S13 and S14). These observations are in agreement with a previous study where it was shown that OsbHLH062 and OsIRO3 regulate the expression of *OsYSL2* and *OsMIR1* independently of the OsIRO2‐OsFIT module (Li et al. 2025). Taken together, these data indicate that OsbHLH064, and more generally IVb bHLH transcription factors, act upstream of the Fe homeostasis regulatory network, orchestrating multiple aspects involved in the regulation of Fe homeostasis, including Fe uptake, Fe translocation, Fe storage and Fe availability signal transduction. Interestingly, it was also shown that OsPRI2 and OsPRI3 are direct activators of *OsYSL2* expression and that the expression of *OsYSL2*, *OsNRAMP1* and *OsMIR* was impaired in *pri1* loss‐of‐function mutants (Zhang et al. 2020). Taken together, these findings support that IVc and IVb bHLHs act as upstream regulators of both regulatory (e.g., *OsIRO2*, *OsIRO3* and *OsIMA1*) and structural genes involved in the maintenance of Fe homeostasis. However, the precise mechanisms by which OsbHLH064 coordinates with other IVc and IVb subgroup bHLHs to integrate and fine‐tune this complex regulatory network will require further investigation.

In summary, our study uncovers OsbHLH064 as a previously uncharacterised IVb subgroup bHLH transcription factor that acts as a central coordinator of Fe homeostasis regulatory networks in rice. Like other IVb members, OsbHLH064 can form heterodimers with IVc subgroup bHLH transcription factors, thereby repressing their transcriptional activation activity and negatively regulating the expression of downstream target genes encoding not only regulatory proteins (e.g., *OsIRO2*, *OsIRO3 and OsIMA1*) but also structural proteins involved in the maintenance of Fe homeostasis (e.g., *OsOPT7*, *OsYSL2* and *OsNRAMP1*). Although OsbHLH064 harbours an EAR‐like motif, it fails to recruit co‐repressors in vivo, indicating that it functions as a passive transcriptional repressor. It likely inhibits target gene expression by competitively binding to shared promoters with IVc bHLH activators, thereby repressing their transcriptional activation. Collectively, these findings establish OsbHLH064 as a central upstream regulator integrating multiple pathways to maintain Fe homeostasis and suggest its potential as a target for biofortification strategies aimed at enhancing iron content in rice grains.

## 4 Materials and Methods

### 4.1 Plant Materials and Growth Conditions

Rice ( *Oryza sativa* L.) cv. Zhonghua 11 (ZH11) was used as wild type (WT). All the mutant and transgenic lines were generated in ZH11 background. Prior to germination, seeds were surface sterilised using 15% NaClO solution for 20 min and then rinsed 3 times with distilled water. Germination was done on humid filter paper for 3 days at 28°C in the dark. For hydroponic culture assays, 3‐day‐old seedlings were transferred to 1/2 Kimura B liquid solution with 100 μM Fe‐EDTA (Yu et al. 2022). Plants were grown in a growth chamber with 70% relative humidity, under a 10 h/14 h (light/dark) light regime at 28°C/22°C (light/dark). The nutrient solution was changed every 2 days. Three‐week‐old seedlings were transferred to 1/2 Kimura B liquid solution without iron supply for iron deficiency treatment for 3–7 days depending on experimental requirements.

WT and transgenic *Nicotiana benthamiana* plants expressing a nuclear‐localised RFP marker were used in this study (Wu et al. 2020). Plants were grown in soil without additional nutrient supply in a greenhouse under a 16 h light / 8 h dark photoperiod at 25°C during the day and 22°C at night.

### 4.2 Generation of Transgenic Plants

Homozygous *OsbHLH064* mutant alleles (ZH11 genetic background) were obtained using CRISPR‐Cas9 gene editing system. The editing vectors were constructed following a previously described protocol (Xie et al. 2014). Briefly, the target site sequence was inserted between the *OsU6a* promoter and *sgRNA* in the *pYLsgRNA‐OsU6a* vector, and then the *OsU6a‐sgRNA* cassette was cloned into the *pYLCRISPR/Cas9Pubi‐H* binary vector prior to rice transformation (Ma et al. 2015). The identification of homozygous mutant lines was confirmed through sequencing analysis. For expression pattern assay, the promoter of *OsbHLH064* (*pOsbHLH064*, 1914 bp prior to the start codon) was amplified and fused to the *GUS* reporter gene and cloned into the *pCAMBIA1300* binary vector for rice transformation. For overexpression of *OsbHLH064*, the *pUbi:OsbHLH064‐Flag* vector was constructed by cloning the open reading frame of *OsbHLH064* into a modified binary expression vector, *Ubi‐1300‐Flag*, downstream of the *UBIQUITIN* promoter. The primers are listed in Table S6.

### 4.3 Yeast Assays

For yeast two‐hybrid assays, the full‐length open reading frame of *OsbHLH064* was cloned into *pGBKT7* (GAL4 binding domain vector), whereas the ORFs of *OsbHLH057*, *OsbHLH058*, *OsbHLH059*, *OsbHLH060*, *OsbHLH061*, *OsbHLH062* and *OsbHLH063* were cloned into *pGADT7* (GAL4 activation domain vector). Yeast transformations were carried out according as described in the Yeast TwoHybrid System User Manual (Clontech). Transformed yeast cells were initially selected on SD/‐Leu/‐Trp (‐LW) medium to confirm successful co‐transformation. Positive colonies were then transferred to SD/‐Leu/‐Trp/‐His/‐Ade (‐LWHA) medium supplemented or not with 1 mM 3‐amino‐1,2,4‐triazole (3‐AT) to assess protein–protein interactions.

Yeast one‐hybrid assays, promoter fragments of potential target genes containing *E‐box* or *G‐box* sequences were cloned into the *pHIS2* vector upstream from the *HIS3* reporter gene. Because the expression of the full‐length *OsbHLH064* in Y187 yeast cells was toxic, a truncated version retaining the N‐terminal DNA‐binding domain was cloned into the *pGADT7* vector. Constructs were co‐transformed into the Y187 yeast strain and selected on SD/‐Leu/‐Trp (‐LW) medium. Protein‐DNA interactions were assessed by yeast growth on SD/‐Leu/‐Trp/‐His (‐LWH) medium supplemented with 3‐AT. Primers used for vector construction are listed in Table S6.

### 4.4 Bimolecular Fluorescence Complementation (BiFC) Assays

BiFC assays were performed as previously reported (Schweiger and Schwenkert 2014). Briefly, the full‐length CDS of *OsbHLH064* was cloned into the *pCAMBIA1300‐nYFP* and *pCAMBIA1300‐cYFP* binary vector, and the CDSs of *OsPRI1*, *OsPRI2*, *OsPRI3*, *OsPRI4*, *OsbHLH061*, *OsbHLH062* and *OsIRO3* were cloned into the *pCAMBIA‐cYFP* vector. *Agrobacterium tumefaciens* (strain GV3101) bacteria containing indicated constructs were co‐infiltrated into *N. benthamiana* leaves and the plants were grown in the dark for 48 h prior to analysis. Epidermal cells were observed by using laser scanning confocal microscopy (Zeiss LSM 880, Germany).

### 4.5 Co‐Immunoprecipitation (Co‐IP) Assays

The full‐length ORF of *OsbHLH064* was cloned into modified *pGreen II 62‐SK* vector with 7 Myc tags under the control of *CaMV 35S* promoter, and the ORFs of *OsPRI1* and *OsbHLH064* were cloned into modified *pGreen II 62‐SK* vector with 3 Flag tags under the control of *CaMV 35S* promoter. All the constructs were transformed into *A. tumefaciens* bacteria, alone or in combination and then infiltrated into *N. benthamiana* leaves. Proteins were extracted and incubated with 10 μL of anti‐Myc Magnetic Beads (Abclonal) at 4°C overnight. The beads were then washed three times with TBS (Tris‐Buffered Saline) buffer and then eluted using SDS‐PAGE sample loading buffer. The OsbHLH064‐Myc and OsbHLHs‐Flag proteins were detected by immunoblotting using anti‐Myc (ABclonal) and anti‐Flag (ABclonal) antibodies, respectively.

### 4.6 Gene Expression Analysis

Total RNA was extracted using a FastPure Plant Total RNA Isolation Kit (Vazyme Biotech, China). The first‐strand cDNA was synthesised using HiScript II Q RT SuperMix for qPCR (+gDNA wiper) (Vazyme Biotech, China) according to the manufacturer's protocol. Real‐Time quantitative reverse transcription PCR (RT‐qPCR) was performed on a CFX Connect Real‐Time PCR Detection System (Bio‐Rad) by using AceQ Universal SYBR qPCR Master Mix (Vazyme Biotech). *OsActin2* was used as a housekeeping gene, and the 2^^(‐ΔΔCt)^ method was used to analyse the relative changes in gene expression between different treatments (Livak and Schmittgen 2001). Three biological replicates were performed for RT‐qPCR analysis. Primers used are listed in Table S6.

### 4.7 Histochemical GUS Assay

Seven‐day‐old seedlings expressing *pOsbHLH064:GUS* gene fusion were transferred to 1/2 Kimura B liquid solution without Fe supply for Fe deficiency treatment for 3 days. Fresh roots were submerged in GUS staining buffer containing 100 mM phosphate solution pH 7.5, 2 mM 5‐bromo‐4‐chloro‐3‐indolyl‐b‐D‐glucuronide (X‐gluc), 0.1% (v/v) Triton X‐100, 10 mM Na_2_‐EDTA, 0.5 mM K_3_[Fe(CN_6_)] and 0.5 mM K_4_[Fe(CN_6_)]. After 30 min vacuum treatment, samples were incubated overnight at 37°C in the dark. Tissues were examined using a stereoscope (MSHOT).

### 4.8 Fluorometric GUS Activity Assay

For quantitative measurement of GUS activity, roots from 7‐day‐old *pOsbHLH064:GUS* seedlings subjected to Fe‐sufficient or Fe‐deficient treatments for 3 days were collected and immediately frozen in liquid nitrogen. Approximately 100 mg of root tissue was homogenised in 400 μL GUS extraction buffer containing 50 mM sodium phosphate (pH 7.0), 10 mM EDTA, 0.1% (v/v) Triton X‐100, 0.1% (v/v) sodium lauryl sarcosinate and 10 mM β‐mercaptoethanol. The homogenates were centrifuged at 12000 *g* for 10 min at 4°C, and the supernatant was used for the assay. GUS activity was determined using 4‐methylumbelliferyl β‐D‐glucuronide (4‐MUG) as the substrate. Reactions were initiated by adding 20 μL protein extract to 180 μL assay buffer containing 1 mM 4‐MUG and incubated at 37°C. The reaction was stopped at designated time points by adding 0.2 M Na₂CO₃, and the released 4‐methylumbelliferone (4‐MU) fluorescence was measured using a microplate reader (excitation 365 nm, emission 456 nm). Protein concentration was determined using Bradford method (Kruger 2009), and GUS activity was expressed as 4‐MU pmol/min/μg protein.

### 4.9 Iron Measurement

After treatments, rice roots and shoots were harvested and rinsed three times with 0.1 M CaCl_2_ solution. Samples were dried at 65°C to constant weight. For each sample, about 100 mg of dry weight sample were digested with 1 mL of nitric acid and hydrogen peroxide solution with a microwave digestion device (Anton Paar Multiwave 3000) at 85°C for 12 h (Gao, Robe, Bettembourg, et al. 2020). Homogenised samples were diluted 5–10 times prior to analysis. Metal concentrations were measured using Inductively Coupled Plasma Mass Spectrometry (ICP‐MS, Agilent 7700 ×).

### 4.10 Chromatin Immunoprecipitation, Followed by Quantitative PCR (ChIP‐qPCR)

For ChIP‐qPCR assays, the *OsbHLH064* overexpression line (OE6) was used. The experiments were performed as previously described with minor modifications (Gao and Dubos 2023). Briefly, approximately 2 *g* of fresh root tissue was harvested and cross‐linked in 1% formaldehyde under vacuum infiltration for 20 min, followed by quenching with 0.125 M glycine for 5 min. Cross‐linked chromatin was extracted and sheared to an average size of 200–500 bp by sonication (Bioruptor). The sheared chromatin was incubated with anti‐Flag M2 antibody (Sigma) at 4°C overnight, followed by immunoprecipitation using protein A/G magnetic beads (Thermofisher) according to the manufacturer's instructions. After reverse cross‐linking, DNA was purified by isopropanol precipitation and subjected to quantitative PCR using gene‐specific primers targeting the promoter regions of putative OsbHLH064 target genes. Enrichment levels were calculated relative to input DNA and normalised to the genomic region where OsbHLH064 is not binding (negative control).

### 4.11 DNA Affinity Purification Sequencing (DAP‐Seq)

OsbHLH064‐Halo proteins were synthesised using Promega TNT SP6 High‐Yield Protein Expression System (Promega) according to the manufacturer's instructions and then purified using anti‐Halo Magnetic Beads (Promega). DAP‐seq was performed as previously described with minor modifications (Bartlett et al. 2017). Briefly, OsbHLH064‐Halo fusion proteins were incubated with sonicated shearing ZH11 rice genomic DNA (200–400 bp) from roots of plants grown under Fe‐sufficient conditions for 2 h and then washed six times with PBS buffer containing 0.005% NP40 and protease inhibitors (MedChemExpress). The bound DNA was eluted in 30 μL pre‐heated EB buffer (10 mM Tris–HCl, pH 8.5). The DNA library preparation and sequencing were conducted by Biorun Technologies Co. Ltd. using the Illumina Hiseq platform. DAP‐seq experiments were performed in two replicates.

### 4.12 RNA‐Seq

Three week old wild type and *osbhlh064‐1* mutant seedlings were transferred to 1/2 Kimura B liquid solution with or without 100 μM Fe‐EDTA for 7 days. Roots were harvested separately for total RNA extraction. RNA sequencing was conducted using the Illumina Hiseq platform. After quality control, clean reads were mapped to *Oryza sativa Japonica* Group (Japanese rice) genome assembly Build 4.0 using Hisat2 tools (Kim et al. 2015). Gene expression levels were estimated by FPKM (fragments per kilobase of transcript per million fragments mapped) using RSEM (Li and Dewey 2011). Differential expression analysis between samples was performed using DESeq2 (Love et al. 2014). Genes with an adjusted *P*‐value < 0.05 and a Fold Change ≥ 2 were assigned as differentially expressed.

### 4.13 Electrophoretic Mobility Shift Assay (EMSA)

Electrophoretic mobility shift assay was performed using a Chemiluminescent EMSA Kit (Coolaber) according to the manufacturer's instructions. The coding sequence of *OsbHLH064* and *OsPRI1* was cloned into the *pCold‐TF* vector for TRIGGER FACTOR (TF) fusion protein production. The TF‐His‐OsbHLH064, TF‐His‐OsPRI1 and TF‐His protein were purified from *Escherichia coli* using His‐tag Protein Purification Kit (Coolaber). The DNA fragments of the target gene promoters containing *G‐Box* or *E‐box* sequences were synthesised and labelled with biotin at the 5′ terminus. Biotin‐unlabelled probes of the same DNA fragments were used as competitors, and the TF‐His protein was used as the negative control. The probe sequences are listed in Table S6.

### 4.14 Transient Luciferase Reporter Assay

*OsbHLH064* and *OsPRI1* were cloned into the *pGreen II 62‐SK* vector under the control of the *CaMV 35S* promoter as effectors. Promoter fragments of *OsIRO2* and *OsIRO3* were inserted into a modified *pGreen0800‐LUC* vector upstream of a *35S* minimal promoter to generate the reporter constructs. All the constructs were transformed into *A. tumefaciens* and then infiltrated alone or in combination into *N. benthamiana* leaves. The Dual‐Luciferase Reporter Assay System (Promega) was used to detect firefly luciferase activity. The Renilla luciferase activity was used as an internal control.

### 4.15 Western Blot

Protein samples were separated on 10% sodium dodecyl‐sulfate polyacrylamide gel. Following separation, proteins were transferred from the gel onto a nitrocellulose membrane. Rabbit monoclonal anti‐Myc (ABclonal) and anti‐Flag (ABclonal) were used as primary antibodies, and HRP‐labelled goat anti‐rabbit IgG (H + L) (ABclonal) was used as a secondary antibody. Target proteins on the membrane were detected by chemiluminescence using ECL Western Blotting Substrate (Beyotime). Signals on the membrane were recorded using a CCD chemiluminescence detection camera.

### 4.16 Reactive Oxygen Species (ROS) and Cell Death Staining

Histochemical staining was performed to detect reactive oxygen species (ROS) and cell death. For hydrogen peroxide (H₂O₂) accumulation, leaves were vacuum‐infiltrated in 1 mg/mL 3,3′‐diaminobenzidine (DAB) solution and incubated at 28°C for 12 h in the dark, then decolorised in 95% ethanol at 80°C until chlorophyll was removed. Superoxide anion (O₂^−^•) was visualised by incubating samples in 0.5 mg/mL nitro blue tetrazolium (NBT) solution at 28°C for 24 h, followed by decolorisation in 95% ethanol. To visualise ROS distribution in leaves, samples were incubated with 10 μM H_2_DCFDA (Abbkine) for 30 min in the dark, washed with phosphate‐buffered saline (PBS) for three times and observed using a confocal laser scanning microscope (LSM880, Zeiss). Trypan blue staining was used to detect cell death by immersing samples in 0.4% trypan blue solution, boiling for 5 min and incubating in the dark for over 24 h before imaging.

### 4.17 Perls/DAB Staining

Rice leaf samples were prepared for Perls/DAB staining by following a protocol based on previous methods (Zhou et al. 2024; Vargas and Roschzttardtz 2023). Briefly, freshly harvested leaves were placed in fixation solution (0.2 M phosphate buffer, pH 7.2, containing 1% (v/v) glutaraldehyde and 2% (v/v) formaldehyde), vacuum‐infiltrated for about 30 min to ensure thorough penetration, and then kept at 4°C for 2–3 days. After fixation, leaf tissues were dehydrated through an ethanol gradient (50%, 60%, 70%, 80%, 90%) and then placed in 100% ethanol overnight to ensure complete dehydration. The samples were then embedded in Technovit 7100 resin (Kulzer) according to the kit instructions, and thin sections of approximately 8 μm thickness were obtained using a microtome (Leica EM UC6). For ferric Fe detection, sections were vacuum‐infiltrated for 5 min with a 1:1 mixture of 4% (v/v) HCl and 4% (w/v) potassium ferrocyanide, then incubated at room temperature for 30 min. To enhance the staining signal, the sections were rinsed thoroughly with distilled water, then incubated for 1 h in methanol containing 10 mM sodium azide (NaN₃) and 0.3% (v/v) hydrogen peroxide (H₂O₂) to block endogenous peroxidase activity. After washing with phosphate‐buffered saline (PBS), the sections were further incubated in a DAB intensification solution (0.025% (w/v) DAB, 0.005% (v/v) H₂O₂, 0.005% (w/v) CoCl₂ and 0.1 M PBS, pH 7.4) for 30 min. The reaction was stopped by rinsing with distilled water. Finally, the stained leaf sections were examined and photographed using a bright‐field microscope (Leica DM6 B).

### 4.18 Statistical Analysis

Statistical analyses of experimental data were performed using Student's *t*‐test or one‐way ANOVA using GRAPAD PRISM (v.10.5.0). RNA‐seq raw sequence data can be found in the NCBI Sequence Read Archive (http://www.ncbi.nlm.nih.gov/sra) under accession no PRJNA1302394.

## Author Contributions

**F.G**. and **D.Z.W**. conceived and supervised the project. **F.G**., **Z.K.Z**, **J.T.Y**, **N.Z**, **Y.Q.D**, **K.X.X**, **J.Z**., **H.L.Z**. and **Z.J.F**. performed experiments. **F.G**. and **Z.K.Z**. analysed the data. **F.G**. drafted the manuscript. **C.D**. and **D.Z.W**. revised and edited the manuscript. **L.H.K**., **T.Y**., **L.L**. and **C.D**. gave comments to the project. All authors proofread and approved the manuscript. **F.G**. and **Z.K.Z**. contributed equally to this work.

## Funding

This work was supported by National Natural Science Foundation of China, 32200227. Science and Technology Innovation Program of Hunan Province, 2024RC1055. Natural Science Foundation of Hunan Province of China, 2023JJ40312, 2023JJ40325, 2026JJ50139. Postgraduate Scientific Research Innovation Project of Hunan Province, CX20251085. The Yuelushan Laboratory Breeding Program, YLS‐2025‐ZY01004. The Scientific Research Fund of Hunan Provincial Education Department (25A0212).

## Conflicts of Interest

The authors declare no conflicts of interest.

## Supporting information


**Figure S1:** Negative control for bimolecular fluorescence complementation (BiFC) assays.
**Figure S2:** Phenotypic analysis of *osbhlh064* knockout mutants.
**Figure S3:** Protein sequence analysis of the *osbhlh064* mutant.
**Figure S4:** Iron concentrations in *osbhlh064* mutant brown seeds.
**Figure S5:** Gene Ontology (GO) enrichment analysis of differentially expressed genes in *osbhlh064‐1* roots compared to wild type under iron‐sufficient and iron‐deficient conditions.
**Figure S6:** Expression analysis of Fe homeostasis‐related genes in the roots of *osbhlh064* mutants.
**Figure S7:** Overlapping transcriptional regulation by OsbHLH064 and OsbHLH061 under Fe‐sufficient conditions.
**Figure S8:** Expression analysis of *OsbHLH064* in T_0_ transgenic lines.
**Figure S9:** Histochemical detection of reactive oxygen species (ROS) in *OsbHLH064*‐overexpressing leaves.
**Figure S10:** GSH contents in *OsbHLH064* overexpression lines.
**Figure S11:** Field performance of *OsbHLH064* overexpressing lines under normal paddy conditions.
**Figure S12:** Expression analysis of Fe homeostasis‐related genes in *OsbHLH064* overexpression lines.
**Figure S13:** OsbHLH064 and OsPRI1 bind to the same promoter regions of target genes in vitro.
**Figure S14:** OsbHLH064 binds to the promoters of Fe homeostasis‐related genes.
**Figure S15:** Tag free OsPRI1 bind to the promoter regions of *OsIRO2* in vitro.
**Figure S16:** Multiple sequence alignment of IVb subgroup bHLH proteins from rice and Arabidopsis
**Figure S17:** OsbHLH064 does not interact with corepressors OsTPR1/2 or OsTPL in yeast.
**Figure S18:** Transactivation activity assay of OsbHLH064 in yeast and tobacco leaves

**Table S1:** Differentially expressed genes (DEGs) identified from RNA‐seq analysis in this study

**Table S2:** Gene Ontology (GO) Enrichment Analysis of Differentially Expressed Genes (DEGs)

**Table S3:** All peaks identified by DAP‐seq analysis

**Table S4:** GO term enrichment (Molecular Function category) for genes bound by OsbHLH064 identified by DAP‐seq

**Table S5:** Overlap between DAP‐seq targets and genes differentially expressed in the mutant under normal or Fe‐deficient conditions

**Table S6:** List of primers used in this study.

## Data Availability

The data that support the findings of this study are available in the Supporting Information for this paper or available from the corresponding author upon reasonable request.
