# Supplementary material for: OsbHLH064, an IVb bHLH Transcription Factor, Regulates Iron Homeostasis and Enhances Grain Fe Accumulation in Rice
Source: Plant Biotechnol J. 2026 Feb 23;24(6):3754–76. doi: 10.1111/pbi.70593 (PMC13205751; doi:10.1111/pbi.70593)
Supplement: Supplementary file 1 — Figure S1: Negative control for bimolecular fluorescence complementation (BiFC) assays. Figure S2: Phenotypic analysis of osbhlh064 knockout mutants. Figure S3: Protein sequence analysis of the osbhlh064 mutant. Figure S4: Iron concentrations in osbhlh064 mutant brown seeds. Figure S5: Gene Ontology (GO) enrichment analysis of differentially expressed genes in osbhlh064‐1 roots compared to wild type under iron‐sufficient and iron‐deficient conditions. Figure S6: Expression analysis of Fe homeostasis‐related genes in the roots of osbhlh064 mutants. Figure S7: Overlapping transcriptional regulation by OsbHLH064 and OsbHLH061 under Fe‐sufficient conditions. Figure S8: Expression analysis of OsbHLH064 in T0 transgenic lines. Figure S9: Histochemical detection of reactive oxygen species (ROS) in OsbHLH064‐overexpressing leaves. Figure S10: GSH contents in OsbHLH064 overexpression lines. Figure S11: Field performance of OsbHLH064 overexpressing lines under normal paddy conditions. Figure S12: Expression analysis of Fe homeostasis‐related genes in OsbHLH064 overexpression lines. Figure S13: OsbHLH064 and OsPRI1 bind to the same promoter regions of target genes in vitro. Figure S14: OsbHLH064 binds to the promoters of Fe homeostasis‐related genes. Figure S15: Tag free OsPRI1 bind to the promoter regions of OsIRO2 in vitro. Figure S16: Multiple sequence alignment of IVb subgroup bHLH proteins from rice and Arabidopsis Figure S17: OsbHLH064 does not interact with corepressors OsTPR1/2 or OsTPL in yeast. Figure S18: Transactivation activity assay of OsbHLH064 in yeast and tobacco leaves [file PBI-24-3754-s002.pptx]

## Slide 1
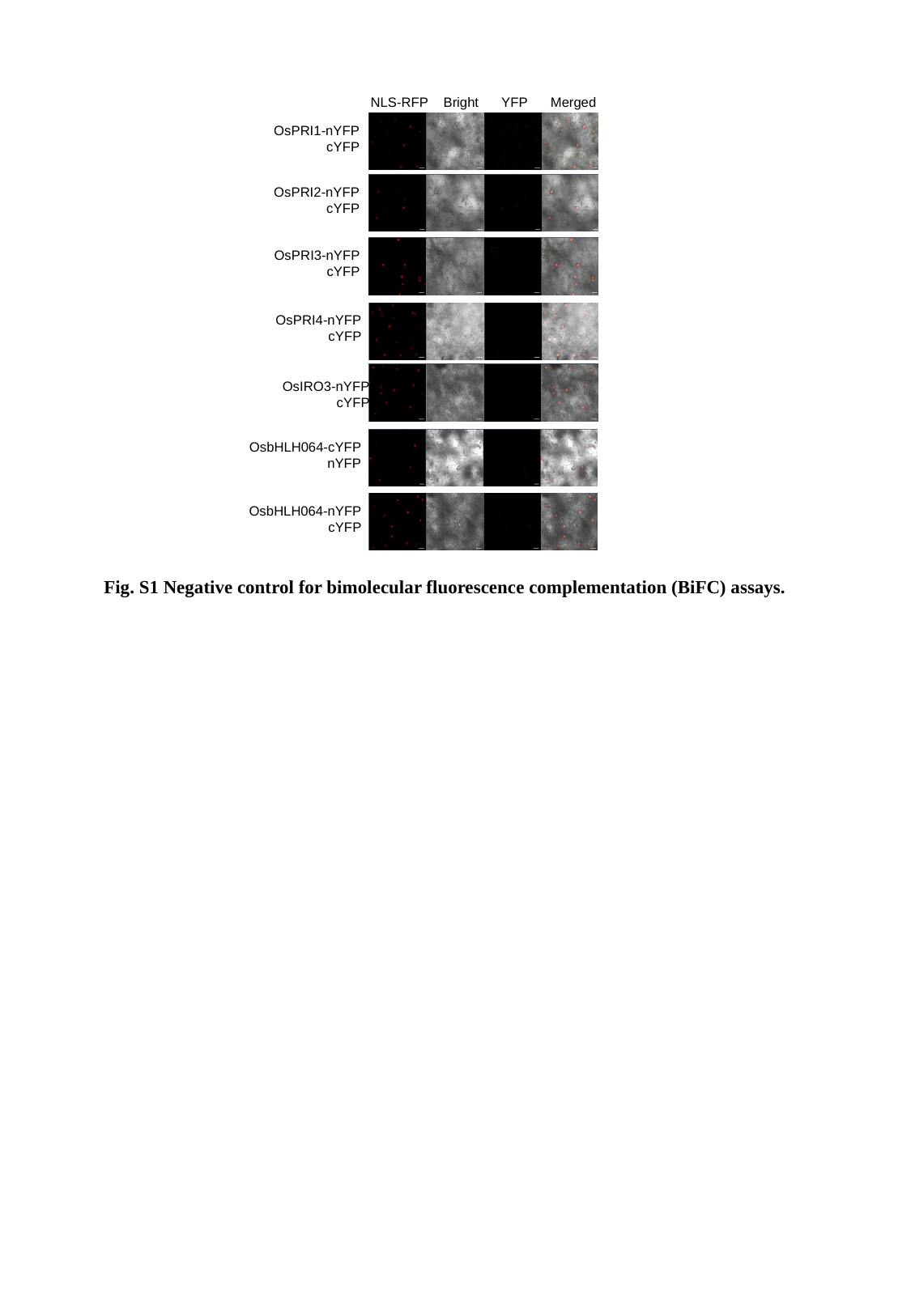

NLS-RFP Bright YFP Merged
OsPRI1-nYFP
cYFP
OsPRI2-nYFP
cYFP
OsPRI3-nYFP
cYFP
OsPRI4-nYFP
cYFP
OsIRO3-nYFP
cYFP
OsbHLH064-cYFP
nYFP
OsbHLH064-nYFP
cYFP
Fig. S1 Negative control for bimolecular fluorescence complementation (BiFC) assays.

## Slide 2
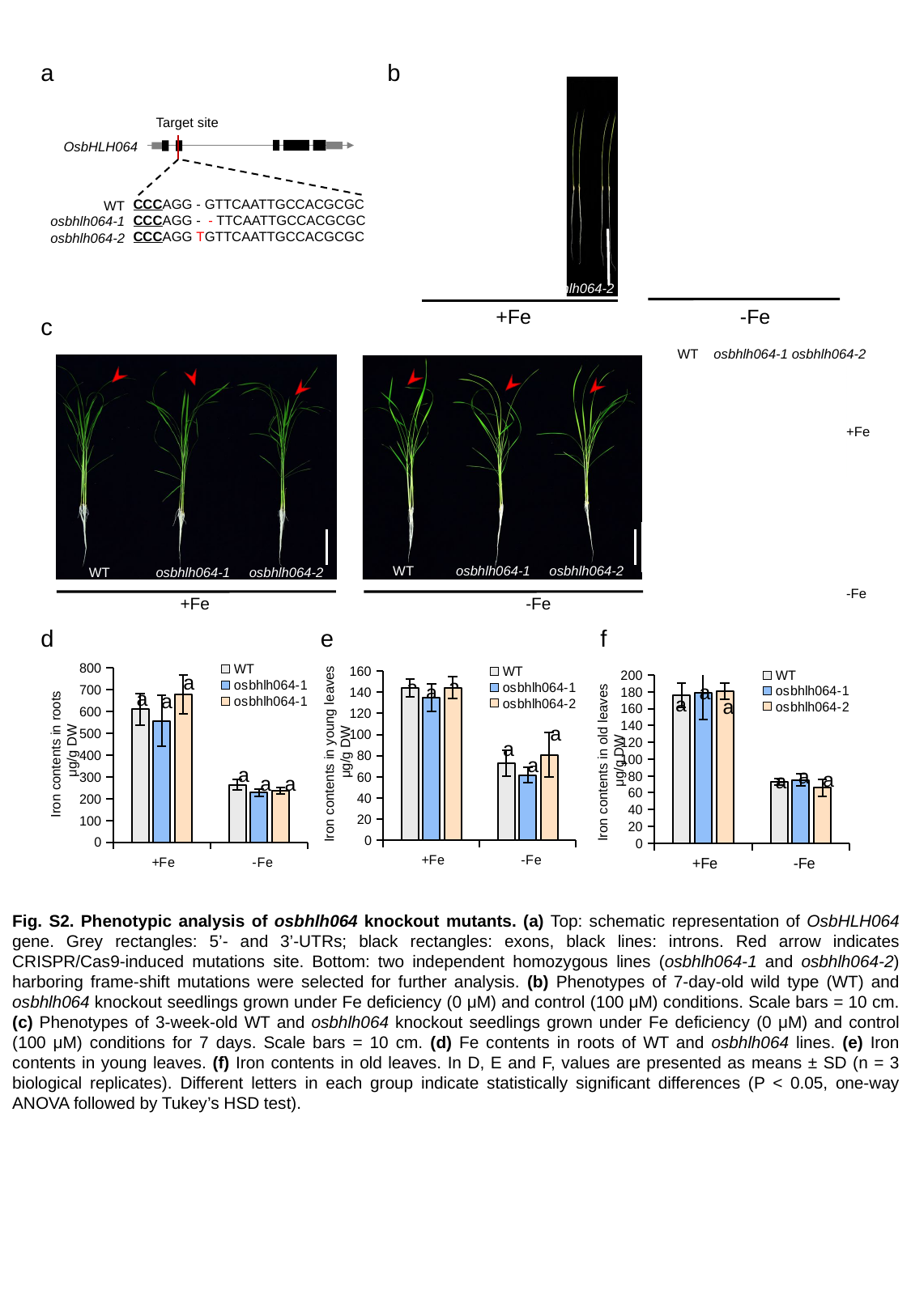

a b
c
d e f
 WT osbhlh064-1 osbhlh064-2
 WT osbhlh064-1 osbhlh064-2
+Fe -Fe
Target site
OsbHLH064
CCCAGG - GTTCAATTGCCACGCGC
CCCAGG - - TTCAATTGCCACGCGC
CCCAGG TGTTCAATTGCCACGCGC
WT
osbhlh064-1
osbhlh064-2
WT osbhlh064-1 osbhlh064-2
+Fe
-Fe
WT osbhlh064-1 osbhlh064-2
WT osbhlh064-1 osbhlh064-2
+Fe -Fe
### Chart
| Category | | osbhlh064-1 | osbhlh064-1 |
|---|---|---|---|
| +Fe | 610.455419716801 | 557.2664615812656 | 679.1315833945432 |
| -Fe | 263.5801579197421 | 227.318252585203 | 235.95039881213216 |a
a
a
Iron contents in roots
μg/g DW
a
a
a
### Chart
| Category | | | |
|---|---|---|---|
| +Fe | 176.304262082162 | 179.00882986358403 | 180.84989335747176 |
| -Fe | 73.15812920239537 | 75.15872494880715 | 65.9144115428307 |a
a
a
Iron contents in old leaves μg/g DW
a
a
a
### Chart
| Category | | | |
|---|---|---|---|
| +Fe | 144.26534340138176 | 135.114932170982 | 144.44359751471873 |
| -Fe | 73.0485900435853 | 61.72157313818896 | 80.62658768545705 |a
a
a
a
Iron contents in young leaves μg/g DW
a
a
Fig. S2. Phenotypic analysis of osbhlh064 knockout mutants. (a) Top: schematic representation of OsbHLH064 gene. Grey rectangles: 5’- and 3’-UTRs; black rectangles: exons, black lines: introns. Red arrow indicates CRISPR/Cas9-induced mutations site. Bottom: two independent homozygous lines (osbhlh064-1 and osbhlh064-2) harboring frame-shift mutations were selected for further analysis. (b) Phenotypes of 7-day-old wild type (WT) and osbhlh064 knockout seedlings grown under Fe deficiency (0 μM) and control (100 μM) conditions. Scale bars = 10 cm. (c) Phenotypes of 3-week-old WT and osbhlh064 knockout seedlings grown under Fe deficiency (0 μM) and control (100 μM) conditions for 7 days. Scale bars = 10 cm. (d) Fe contents in roots of WT and osbhlh064 lines. (e) Iron contents in young leaves. (f) Iron contents in old leaves. In D, E and F, values are presented as means ± SD (n = 3 biological replicates). Different letters in each group indicate statistically significant differences (P < 0.05, one-way ANOVA followed by Tukey’s HSD test).

## Slide 3
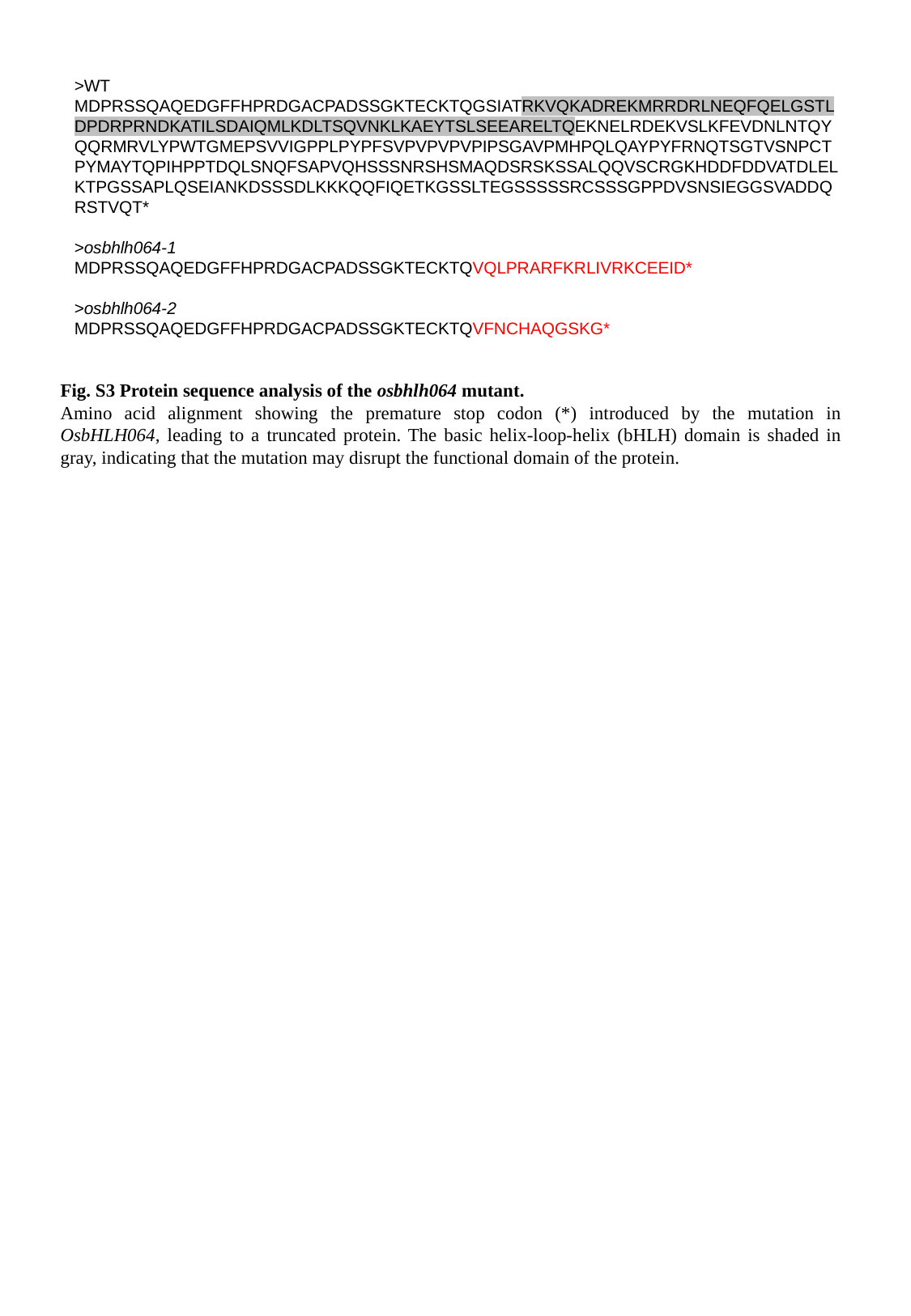

>WT
MDPRSSQAQEDGFFHPRDGACPADSSGKTECKTQGSIATRKVQKADREKMRRDRLNEQFQELGSTLDPDRPRNDKATILSDAIQMLKDLTSQVNKLKAEYTSLSEEARELTQEKNELRDEKVSLKFEVDNLNTQYQQRMRVLYPWTGMEPSVVIGPPLPYPFSVPVPVPVPIPSGAVPMHPQLQAYPYFRNQTSGTVSNPCTPYMAYTQPIHPPTDQLSNQFSAPVQHSSSNRSHSMAQDSRSKSSALQQVSCRGKHDDFDDVATDLELKTPGSSAPLQSEIANKDSSSDLKKKQQFIQETKGSSLTEGSSSSSRCSSSGPPDVSNSIEGGSVADDQRSTVQT*
>osbhlh064-1
MDPRSSQAQEDGFFHPRDGACPADSSGKTECKTQVQLPRARFKRLIVRKCEEID*
>osbhlh064-2
MDPRSSQAQEDGFFHPRDGACPADSSGKTECKTQVFNCHAQGSKG*
Fig. S3 Protein sequence analysis of the osbhlh064 mutant.
Amino acid alignment showing the premature stop codon (*) introduced by the mutation in OsbHLH064, leading to a truncated protein. The basic helix-loop-helix (bHLH) domain is shaded in gray, indicating that the mutation may disrupt the functional domain of the protein.

## Slide 4
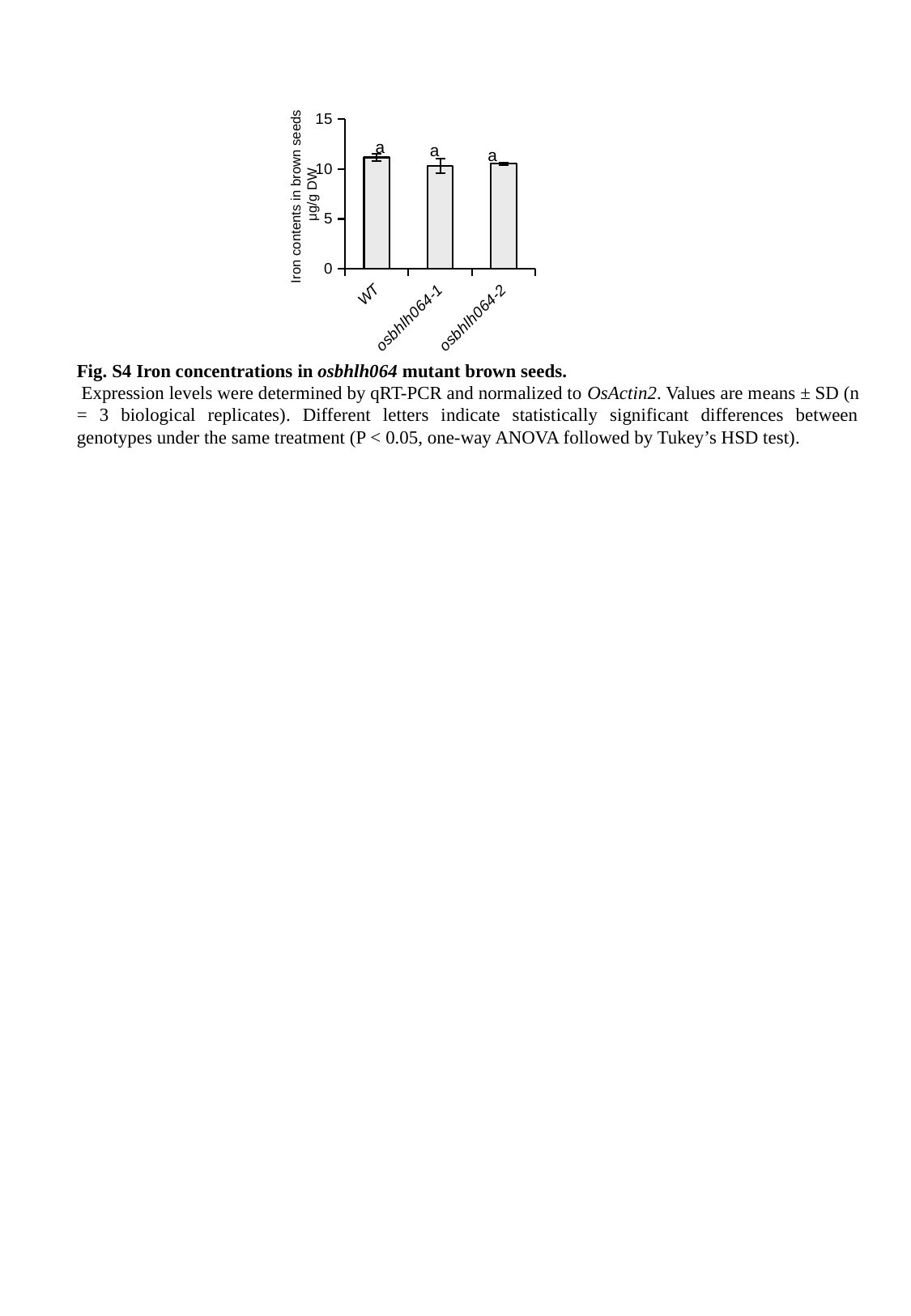

### Chart
| Category | |
|---|---|
| WT | 11.142339768710768 |
| osbhlh064-1 | 10.30788917284289 |
| osbhlh064-2 | 10.501933649957401 |a
a
a
Iron contents in brown seeds μg/g DW
Fig. S4 Iron concentrations in osbhlh064 mutant brown seeds.
 Expression levels were determined by qRT-PCR and normalized to OsActin2. Values are means ± SD (n = 3 biological replicates). Different letters indicate statistically significant differences between genotypes under the same treatment (P < 0.05, one-way ANOVA followed by Tukey’s HSD test).

## Slide 5
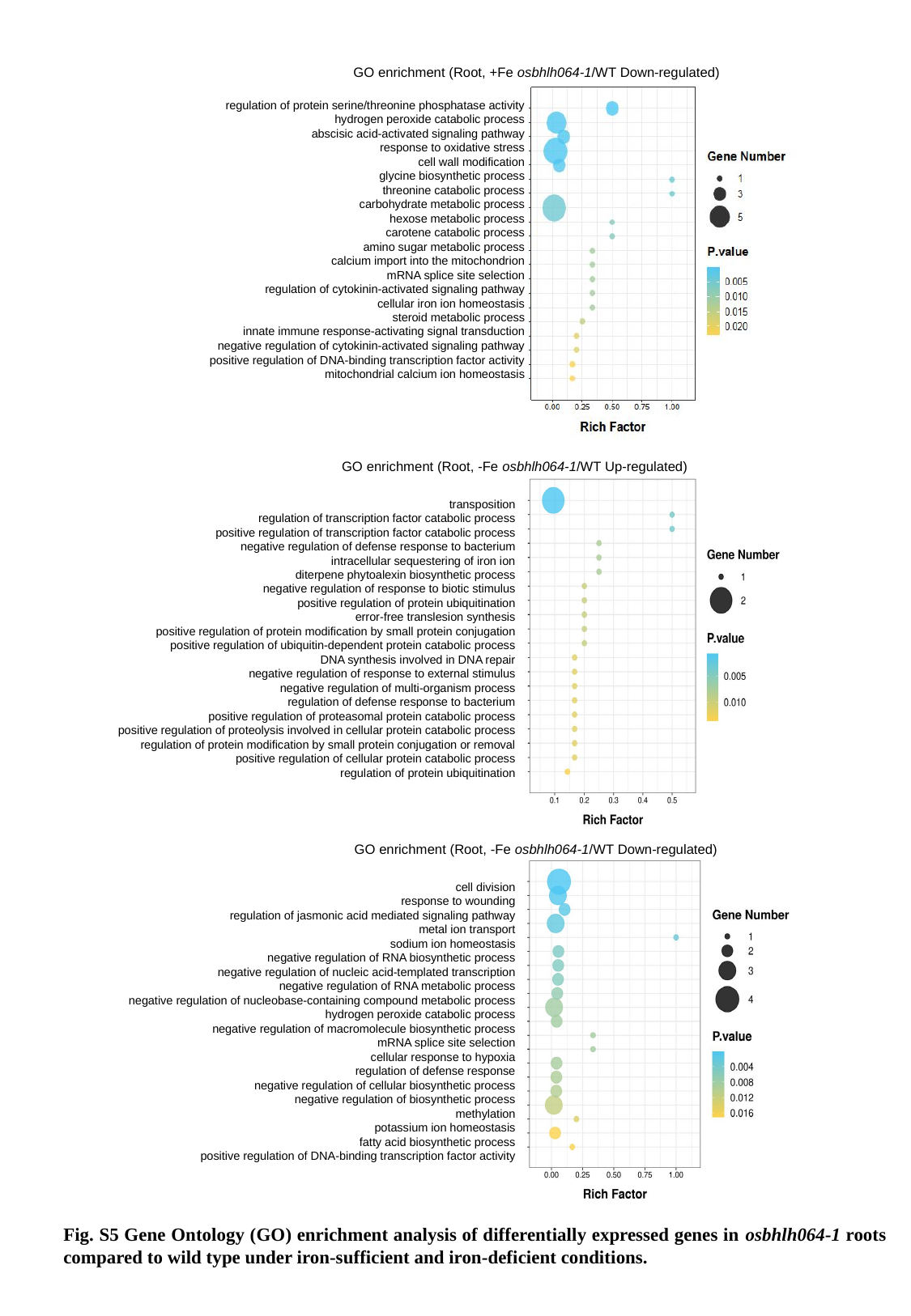

GO enrichment (Root, +Fe osbhlh064-1/WT Down-regulated)
 regulation of protein serine/threonine phosphatase activity
 hydrogen peroxide catabolic process
 abscisic acid-activated signaling pathway
 response to oxidative stress
 cell wall modification
 glycine biosynthetic process
 threonine catabolic process
 carbohydrate metabolic process
 hexose metabolic process
 carotene catabolic process
 amino sugar metabolic process
 calcium import into the mitochondrion
 mRNA splice site selection
 regulation of cytokinin-activated signaling pathway
 cellular iron ion homeostasis
 steroid metabolic process
 innate immune response-activating signal transduction
 negative regulation of cytokinin-activated signaling pathway
 positive regulation of DNA-binding transcription factor activity
 mitochondrial calcium ion homeostasis
GO enrichment (Root, -Fe osbhlh064-1/WT Up-regulated)
 transposition
 regulation of transcription factor catabolic process
 positive regulation of transcription factor catabolic process
 negative regulation of defense response to bacterium
 intracellular sequestering of iron ion
 diterpene phytoalexin biosynthetic process
 negative regulation of response to biotic stimulus
 positive regulation of protein ubiquitination
 error-free translesion synthesis
 positive regulation of protein modification by small protein conjugation
 positive regulation of ubiquitin-dependent protein catabolic process
 DNA synthesis involved in DNA repair
 negative regulation of response to external stimulus
 negative regulation of multi-organism process
 regulation of defense response to bacterium
 positive regulation of proteasomal protein catabolic process
 positive regulation of proteolysis involved in cellular protein catabolic process
 regulation of protein modification by small protein conjugation or removal
 positive regulation of cellular protein catabolic process
 regulation of protein ubiquitination
GO enrichment (Root, -Fe osbhlh064-1/WT Down-regulated)
 cell division
 response to wounding
 regulation of jasmonic acid mediated signaling pathway
 metal ion transport
 sodium ion homeostasis
 negative regulation of RNA biosynthetic process
 negative regulation of nucleic acid-templated transcription
 negative regulation of RNA metabolic process
 negative regulation of nucleobase-containing compound metabolic process
 hydrogen peroxide catabolic process
 negative regulation of macromolecule biosynthetic process
 mRNA splice site selection
 cellular response to hypoxia
 regulation of defense response
 negative regulation of cellular biosynthetic process
 negative regulation of biosynthetic process
 methylation
 potassium ion homeostasis
 fatty acid biosynthetic process
 positive regulation of DNA-binding transcription factor activity
Fig. S5 Gene Ontology (GO) enrichment analysis of differentially expressed genes in osbhlh064-1 roots compared to wild type under iron-sufficient and iron-deficient conditions.

## Slide 6
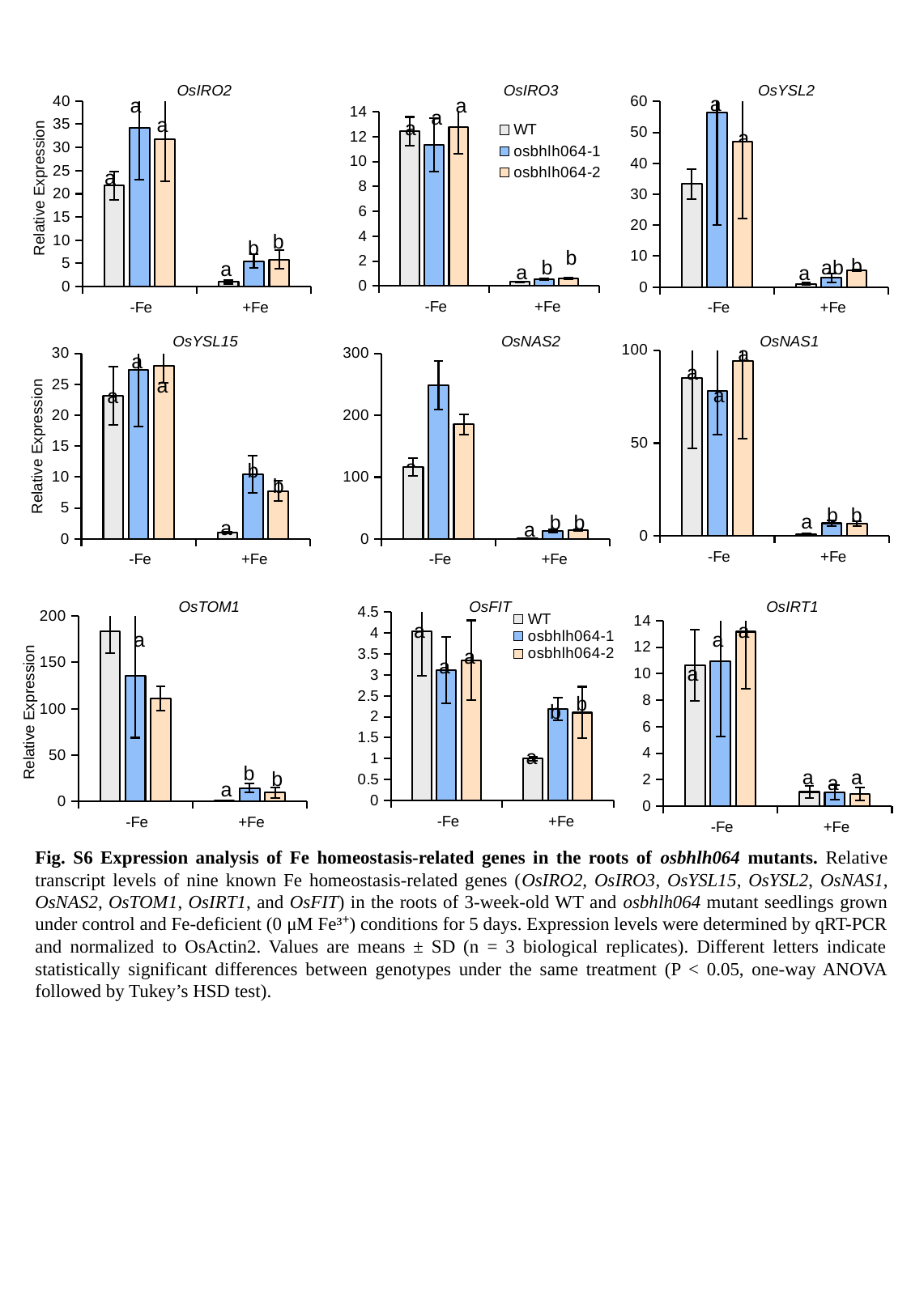

OsIRO2 OsIRO3 OsYSL2
### Chart
| Category | | | |
|---|---|---|---|
| -Fe | 21.74574348995134 | 34.19511514019304 | 31.755005843749778 |
| +Fe | 1.0549571965805393 | 5.472123003146356 | 5.843913203439078 |
### Chart
| Category | | | |
|---|---|---|---|
| -Fe | 33.29231833916854 | 56.54354197832729 | 47.0248647916241 |
| +Fe | 1.029919755855379 | 2.98834449043099 | 5.311189596655194 |a
a
a
b
ab
a
a
a
a
a
b
b
a
### Chart
| Category | | osbhlh064-1 | osbhlh064-2 |
|---|---|---|---|
| -Fe | 12.441349491495536 | 11.331366600326998 | 12.764721188040417 |
| +Fe | 0.32048043603078025 | 0.5421527183837842 | 0.614736697195687 |a
a
Relative Expression
b
b
a
OsYSL15 OsNAS2 OsNAS1
### Chart
| Category | | | |
|---|---|---|---|
| -Fe | 85.0395763344261 | 78.15941468689742 | 94.12501012147136 |
| +Fe | 1.0321673420972846 | 6.811687836755309 | 6.4575518729527674 |a
a
a
b
b
a
### Chart
| Category | | | |
|---|---|---|---|
| -Fe | 23.14937810538684 | 27.34809361568396 | 27.92635010868764 |
| +Fe | 1.004729793715857 | 10.432963949705801 | 7.72772033688859 |
### Chart
| Category | | | |
|---|---|---|---|
| -Fe | 116.53458787766816 | 248.1464768387369 | 185.3391143710295 |
| +Fe | 1.0524057193064686 | 12.52017368685672 | 14.399172228417354 |a
a
a
b
b
a
b
b
a
b
b
a
Relative Expression
OsTOM1 OsFIT OsIRT1
### Chart
| Category | | osbhlh064-1 | osbhlh064-2 |
|---|---|---|---|
| -Fe | 4.040851182199411 | 3.112945781979851 | 3.346987138581189 |
| +Fe | 1.0008026617490808 | 2.1854456496319874 | 2.098782402484151 |
### Chart
| Category | | | |
|---|---|---|---|
| -Fe | 183.4693901162031 | 135.73396938651564 | 111.23638195571374 |
| +Fe | 1.0780528746107967 | 14.572566067371179 | 9.610055804973785 |
### Chart
| Category | | | |
|---|---|---|---|
| -Fe | 10.634024297261826 | 10.95390585217643 | 13.164892624254877 |
| +Fe | 1.0773003032602237 | 1.0224951140718088 | 0.9379272603634377 |a
a
a
a
a
a
a
a
a
a
b
b
a
a
a
b
b
Relative Expression
a
Fig. S6 Expression analysis of Fe homeostasis-related genes in the roots of osbhlh064 mutants. Relative transcript levels of nine known Fe homeostasis-related genes (OsIRO2, OsIRO3, OsYSL15, OsYSL2, OsNAS1, OsNAS2, OsTOM1, OsIRT1, and OsFIT) in the roots of 3-week-old WT and osbhlh064 mutant seedlings grown under control and Fe-deficient (0 μM Fe³⁺) conditions for 5 days. Expression levels were determined by qRT-PCR and normalized to OsActin2. Values are means ± SD (n = 3 biological replicates). Different letters indicate statistically significant differences between genotypes under the same treatment (P < 0.05, one-way ANOVA followed by Tukey’s HSD test).

## Slide 7
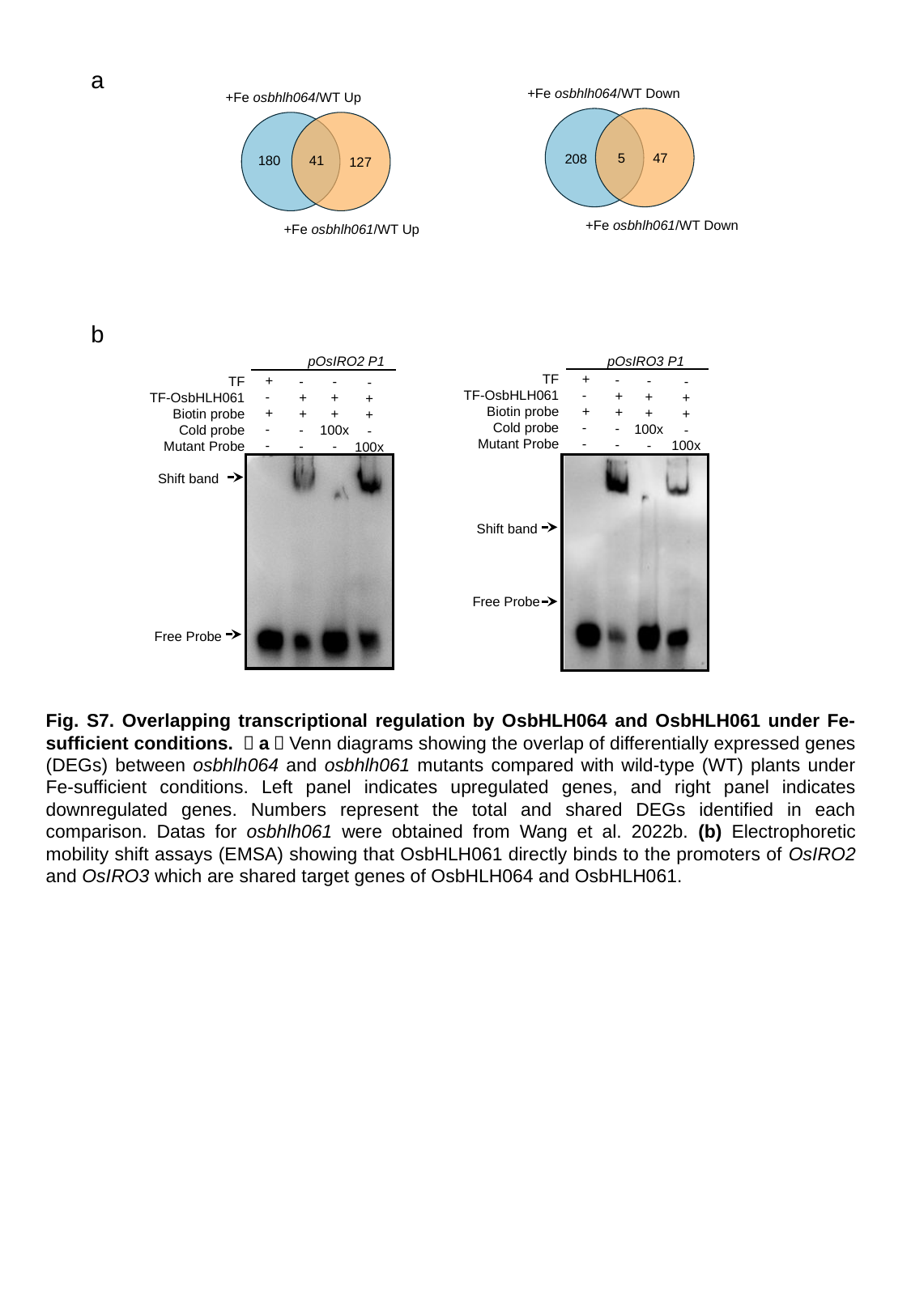

a
b
+Fe osbhlh064/WT Down
5
47
208
+Fe osbhlh061/WT Down
+Fe osbhlh064/WT Up
180
41
127
+Fe osbhlh061/WT Up
pOsIRO2 P1 pOsIRO3 P1
TF
TF-OsbHLH061
Biotin probe
Cold probe
Mutant Probe
+
-
+
-
-
-
+
+
-
-
-
+
+
100x
-
-
+
+
-
100x
Shift band
Free Probe
+
-
+
-
-
-
+
+
-
-
TF
TF-OsbHLH061
Biotin probe
Cold probe
Mutant Probe
-
+
+
100x
-
-
+
+
-
100x
Shift band
Free Probe
Fig. S7. Overlapping transcriptional regulation by OsbHLH064 and OsbHLH061 under Fe-sufficient conditions. （a）Venn diagrams showing the overlap of differentially expressed genes (DEGs) between osbhlh064 and osbhlh061 mutants compared with wild-type (WT) plants under Fe-sufficient conditions. Left panel indicates upregulated genes, and right panel indicates downregulated genes. Numbers represent the total and shared DEGs identified in each comparison. Datas for osbhlh061 were obtained from Wang et al. 2022b. (b) Electrophoretic mobility shift assays (EMSA) showing that OsbHLH061 directly binds to the promoters of OsIRO2 and OsIRO3 which are shared target genes of OsbHLH064 and OsbHLH061.

## Slide 8
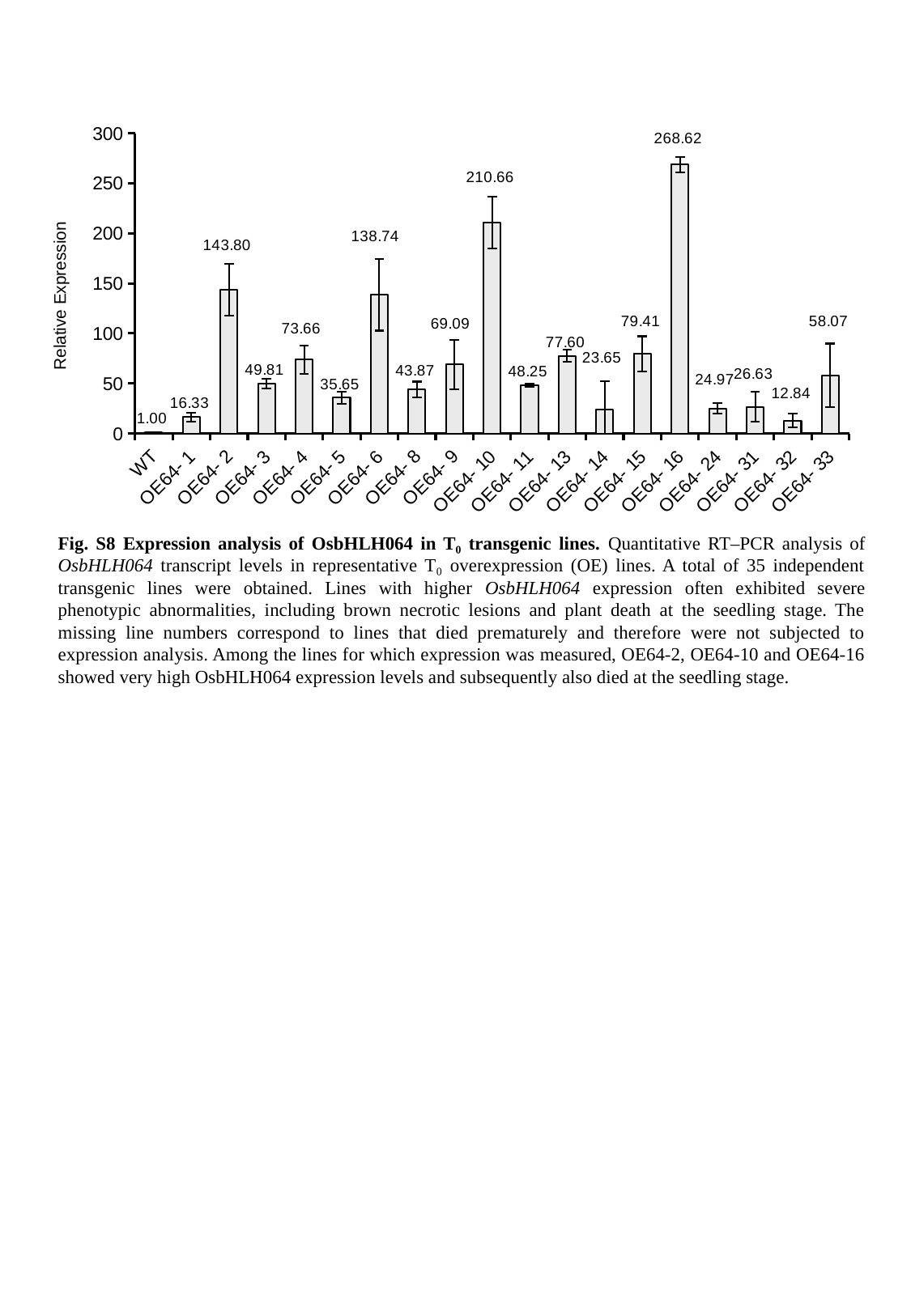

### Chart
| Category | |
|---|---|
| WT | 1.0044387414473146 |
| OE64- 1 | 16.32924882362877 |
| OE64- 2 | 143.79884530520502 |
| OE64- 3 | 49.81110303964292 |
| OE64- 4 | 73.66425660491234 |
| OE64- 5 | 35.645952065617564 |
| OE64- 6 | 138.73937933663305 |
| OE64- 8 | 43.87012535473759 |
| OE64- 9 | 69.09111972467751 |
| OE64- 10 | 210.6552835395832 |
| OE64- 11 | 48.24936104173176 |
| OE64- 13 | 77.59871468064156 |
| OE64- 14 | 23.652045315166124 |
| OE64- 15 | 79.40550907262867 |
| OE64- 16 | 268.61624183912085 |
| OE64- 24 | 24.965657474150817 |
| OE64- 31 | 26.62950324691394 |
| OE64- 32 | 12.837381431742473 |
| OE64- 33 | 58.069433581939336 |Relative Expression
Fig. S8 Expression analysis of OsbHLH064 in T₀ transgenic lines. Quantitative RT–PCR analysis of OsbHLH064 transcript levels in representative T₀ overexpression (OE) lines. A total of 35 independent transgenic lines were obtained. Lines with higher OsbHLH064 expression often exhibited severe phenotypic abnormalities, including brown necrotic lesions and plant death at the seedling stage. The missing line numbers correspond to lines that died prematurely and therefore were not subjected to expression analysis. Among the lines for which expression was measured, OE64-2, OE64-10 and OE64-16 showed very high OsbHLH064 expression levels and subsequently also died at the seedling stage.

## Slide 9
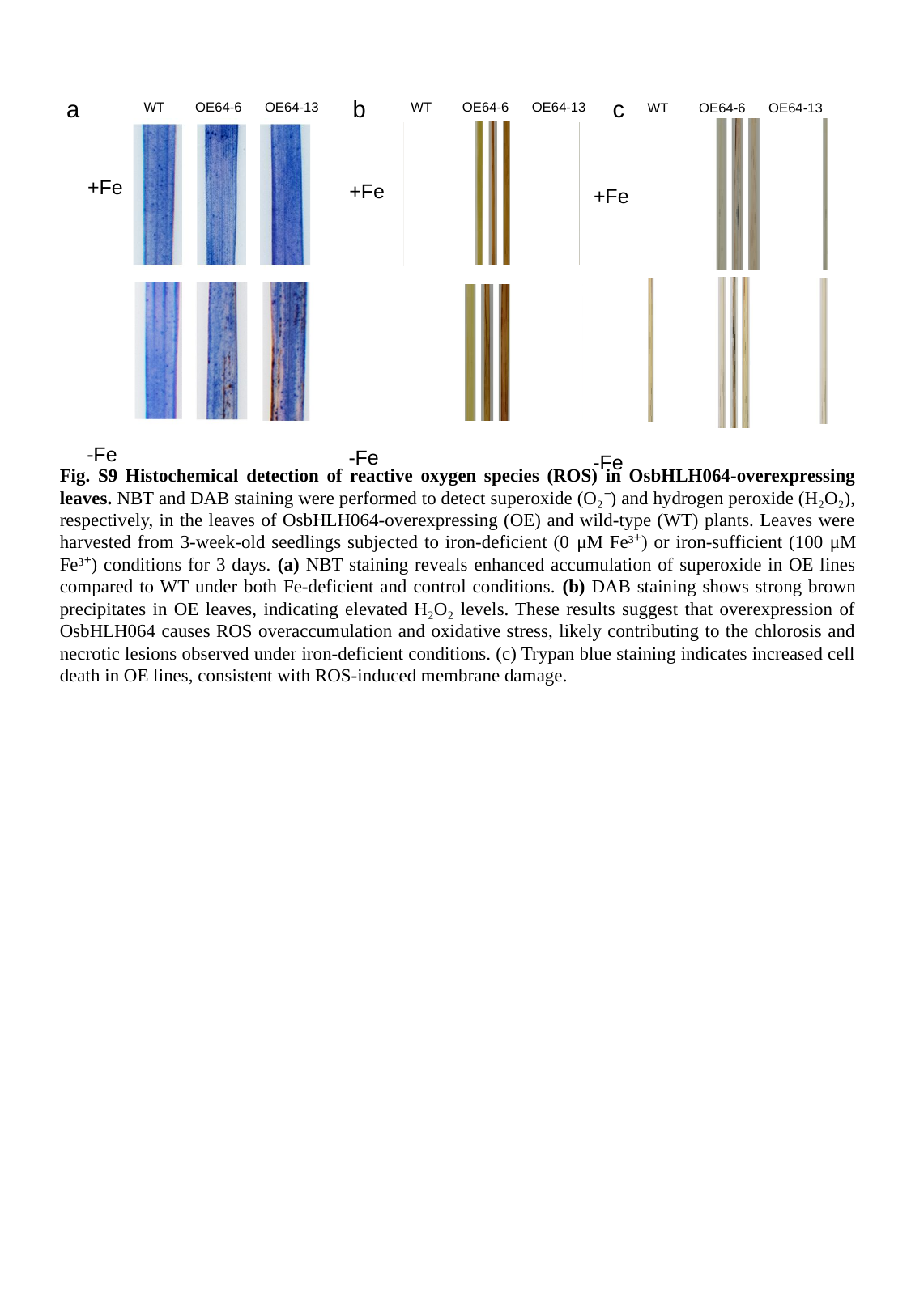

a b c
WT OE64-6 OE64-13
+Fe
-Fe
WT OE64-6 OE64-13
WT OE64-6 OE64-13
+Fe
-Fe
+Fe
-Fe
Fig. S9 Histochemical detection of reactive oxygen species (ROS) in OsbHLH064-overexpressing leaves. NBT and DAB staining were performed to detect superoxide (O₂⁻) and hydrogen peroxide (H₂O₂), respectively, in the leaves of OsbHLH064-overexpressing (OE) and wild-type (WT) plants. Leaves were harvested from 3-week-old seedlings subjected to iron-deficient (0 μM Fe³⁺) or iron-sufficient (100 μM Fe³⁺) conditions for 3 days. (a) NBT staining reveals enhanced accumulation of superoxide in OE lines compared to WT under both Fe-deficient and control conditions. (b) DAB staining shows strong brown precipitates in OE leaves, indicating elevated H₂O₂ levels. These results suggest that overexpression of OsbHLH064 causes ROS overaccumulation and oxidative stress, likely contributing to the chlorosis and necrotic lesions observed under iron-deficient conditions. (c) Trypan blue staining indicates increased cell death in OE lines, consistent with ROS-induced membrane damage.

## Slide 10
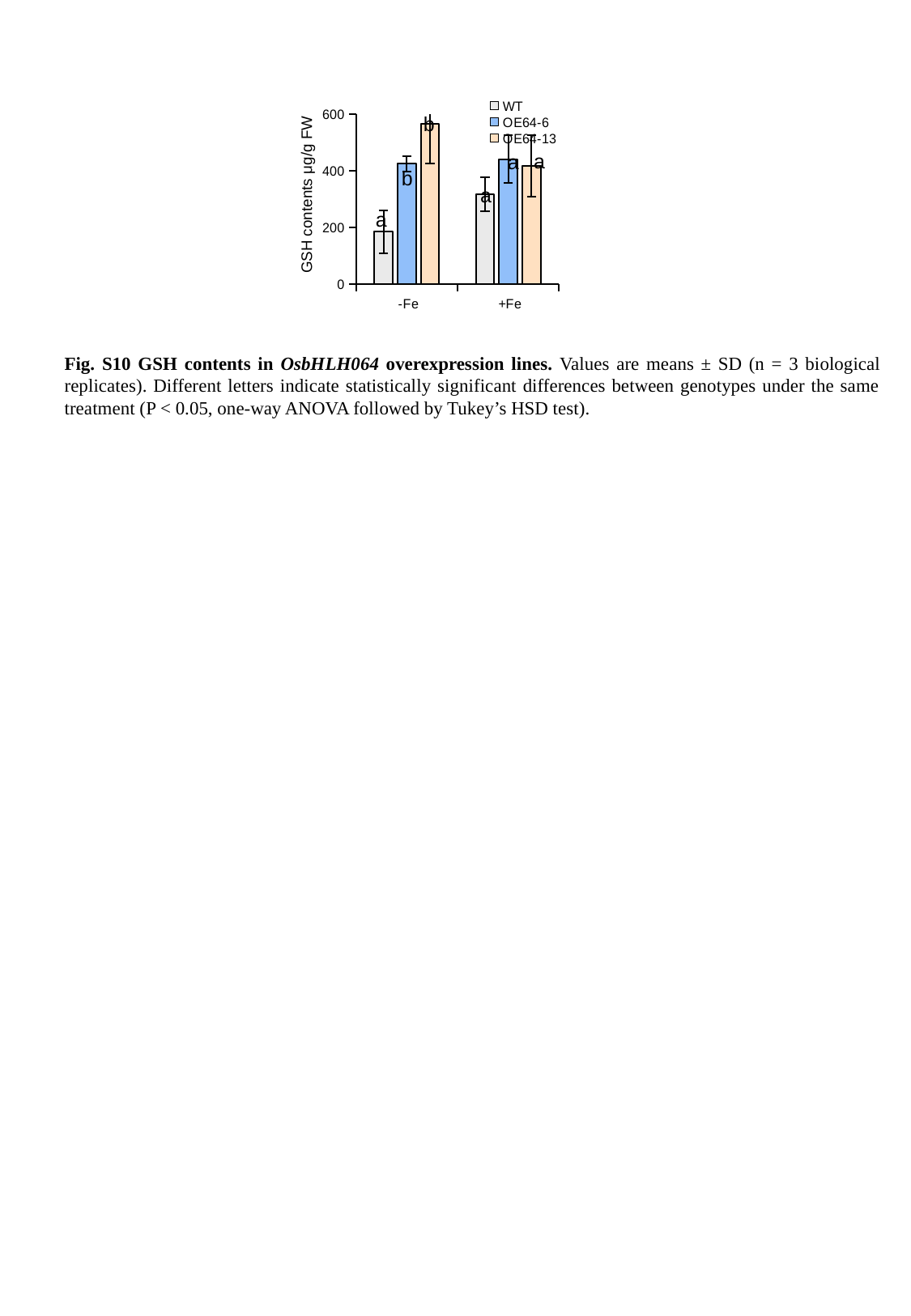

### Chart
| Category | | | |
|---|---|---|---|
| -Fe | 185.22133203138148 | 425.66811226194704 | 568.0400762644466 |
| +Fe | 317.2150852832192 | 441.6500462684354 | 418.23626460704503 |GSH contents μg/g FW
b
a
a
b
a
a
Fig. S10 GSH contents in OsbHLH064 overexpression lines. Values are means ± SD (n = 3 biological replicates). Different letters indicate statistically significant differences between genotypes under the same treatment (P < 0.05, one-way ANOVA followed by Tukey’s HSD test).

## Slide 11
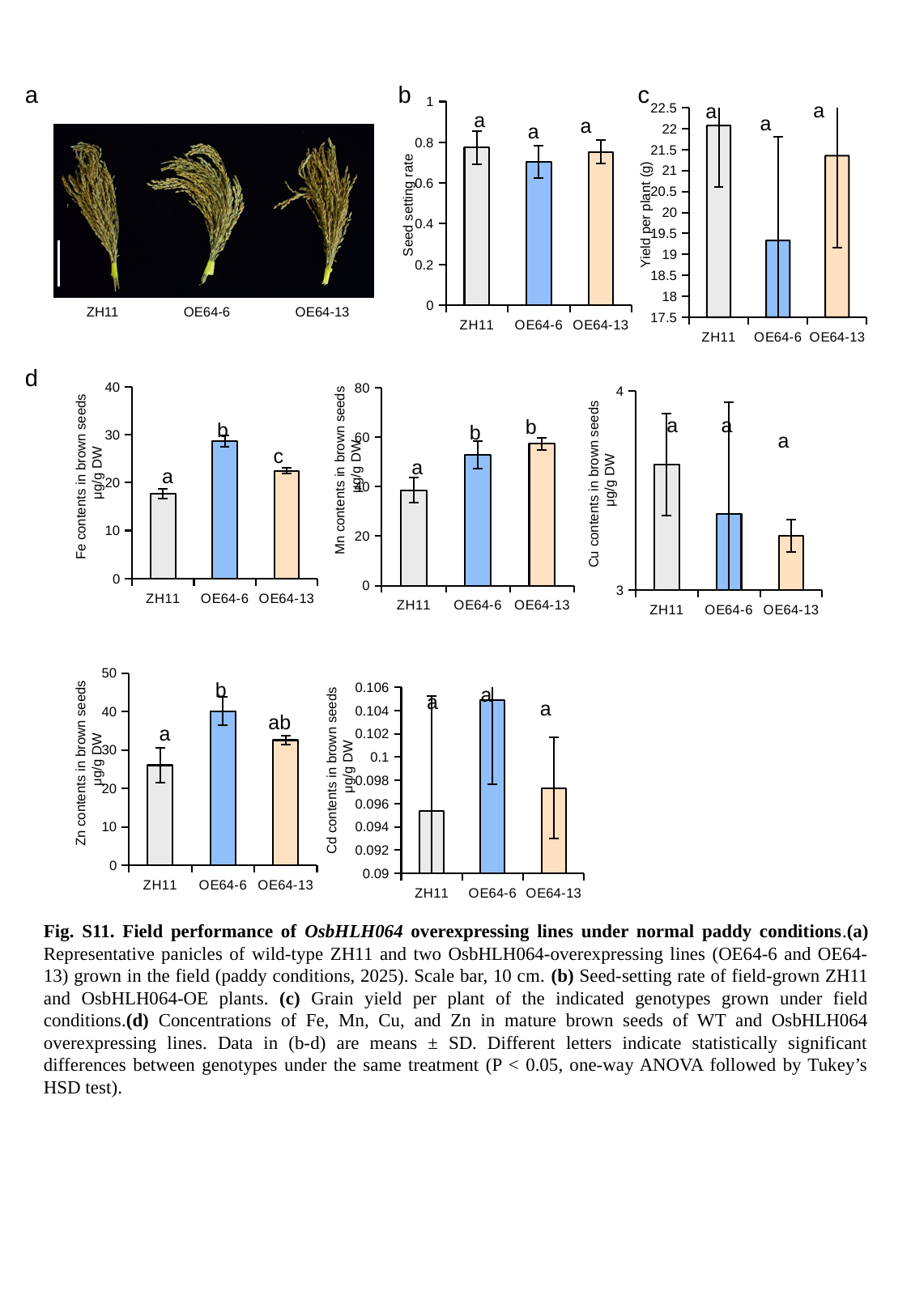

a b c
d
### Chart
| Category | |
|---|---|
| ZH11 | 0.7740780106289274 |
| OE64-6 | 0.7046344641083915 |
| OE64-13 | 0.7531512991595863 |a
a
a
Seed setting rate
a
a
### Chart
| Category | |
|---|---|
| ZH11 | 22.071428571428573 |
| OE64-6 | 19.332857142857144 |
| OE64-13 | 21.35285714285714 |Yield per plant (g)
a
ZH11 OE64-6 OE64-13
### Chart
| Category | |
|---|---|
| ZH11 | 38.54871261015052 |
| OE64-6 | 52.70575742017956 |
| OE64-13 | 57.23019433627314 |Mn contents in brown seeds
μg/g DW
b
b
a
### Chart
| Category | |
|---|---|
| ZH11 | 17.653128795206683 |
| OE64-6 | 28.708047547541724 |
| OE64-13 | 22.50231809271389 |Fe contents in brown seeds
μg/g DW
b
c
a
### Chart
| Category | |
|---|---|
| ZH11 | 3.6293926238951446 |
| OE64-6 | 3.382802037274546 |
| OE64-13 | 3.271093714862134 |Cu contents in brown seeds
 μg/g DW
a
a
a
### Chart
| Category | |
|---|---|
| ZH11 | 26.01603361334537 |
| OE64-6 | 40.06863906567204 |
| OE64-13 | 32.541819460603115 |Zn contents in brown seeds
μg/g DW
b
ab
a
### Chart
| Category | |
|---|---|
| ZH11 | 0.09536548308832277 |
| OE64-6 | 0.10487930765118543 |
| OE64-13 | 0.09734035272154858 |a
a
a
Cd contents in brown seeds
μg/g DW
Fig. S11. Field performance of OsbHLH064 overexpressing lines under normal paddy conditions.(a) Representative panicles of wild-type ZH11 and two OsbHLH064-overexpressing lines (OE64-6 and OE64-13) grown in the field (paddy conditions, 2025). Scale bar, 10 cm. (b) Seed-setting rate of field-grown ZH11 and OsbHLH064-OE plants. (c) Grain yield per plant of the indicated genotypes grown under field conditions.(d) Concentrations of Fe, Mn, Cu, and Zn in mature brown seeds of WT and OsbHLH064 overexpressing lines. Data in (b-d) are means ± SD. Different letters indicate statistically significant differences between genotypes under the same treatment (P < 0.05, one-way ANOVA followed by Tukey’s HSD test).

## Slide 12
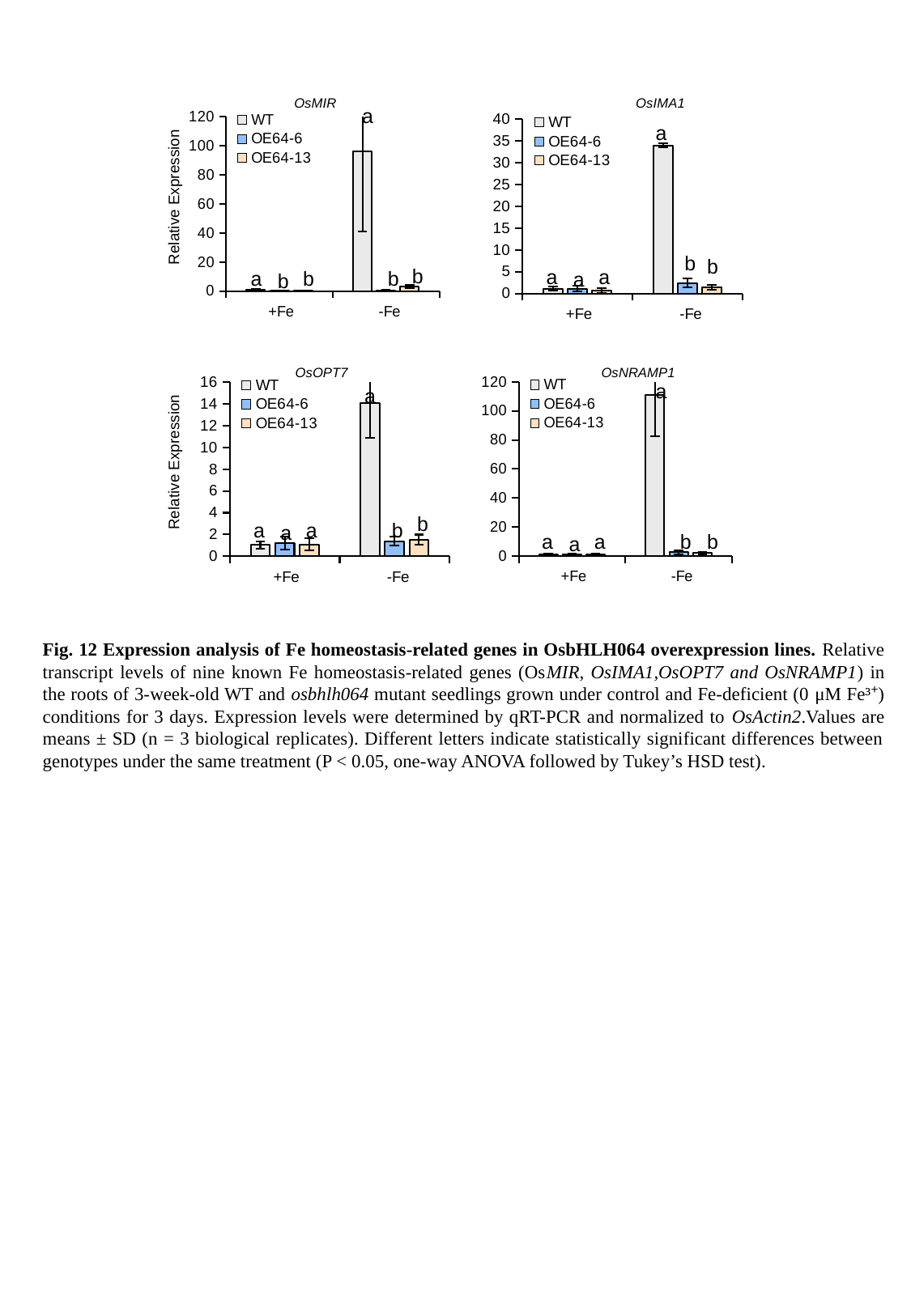

OsMIR OsIMA1
a
### Chart
| Category | | | |
|---|---|---|---|
| +Fe | 1.07138753549252 | 0.038821922397753096 | 0.010264305656851635 |
| -Fe | 96.1977616300539 | 0.360581877114479 | 3.16880421098138 |Relative Expression
b
b
b
a
b
### Chart
| Category | | | |
|---|---|---|---|
| +Fe | 1.0722513990789395 | 1.0974442014187897 | 0.6003494340674362 |
| -Fe | 33.99749192192192 | 2.372616512969685 | 1.3980910459053681 |a
b
b
a
a
a
OsOPT7 OsNRAMP1
### Chart
| Category | | | |
|---|---|---|---|
| +Fe | 1.1145322730627196 | 1.1528936985515643 | 0.8577597405996024 |
| -Fe | 111.04654179909585 | 2.7058050731831678 | 1.9763712685371597 |a
a
b
a
b
a
### Chart
| Category | | | |
|---|---|---|---|
| +Fe | 1.0341223081577537 | 1.2020345782265225 | 1.0924531081249675 |
| -Fe | 14.063629694377942 | 1.3850826392902877 | 1.508005055260583 |a
Relative Expression
b
a
b
a
a
Fig. 12 Expression analysis of Fe homeostasis-related genes in OsbHLH064 overexpression lines. Relative transcript levels of nine known Fe homeostasis-related genes (OsMIR, OsIMA1,OsOPT7 and OsNRAMP1) in the roots of 3-week-old WT and osbhlh064 mutant seedlings grown under control and Fe-deficient (0 μM Fe³⁺) conditions for 3 days. Expression levels were determined by qRT-PCR and normalized to OsActin2.Values are means ± SD (n = 3 biological replicates). Different letters indicate statistically significant differences between genotypes under the same treatment (P < 0.05, one-way ANOVA followed by Tukey’s HSD test).

## Slide 13
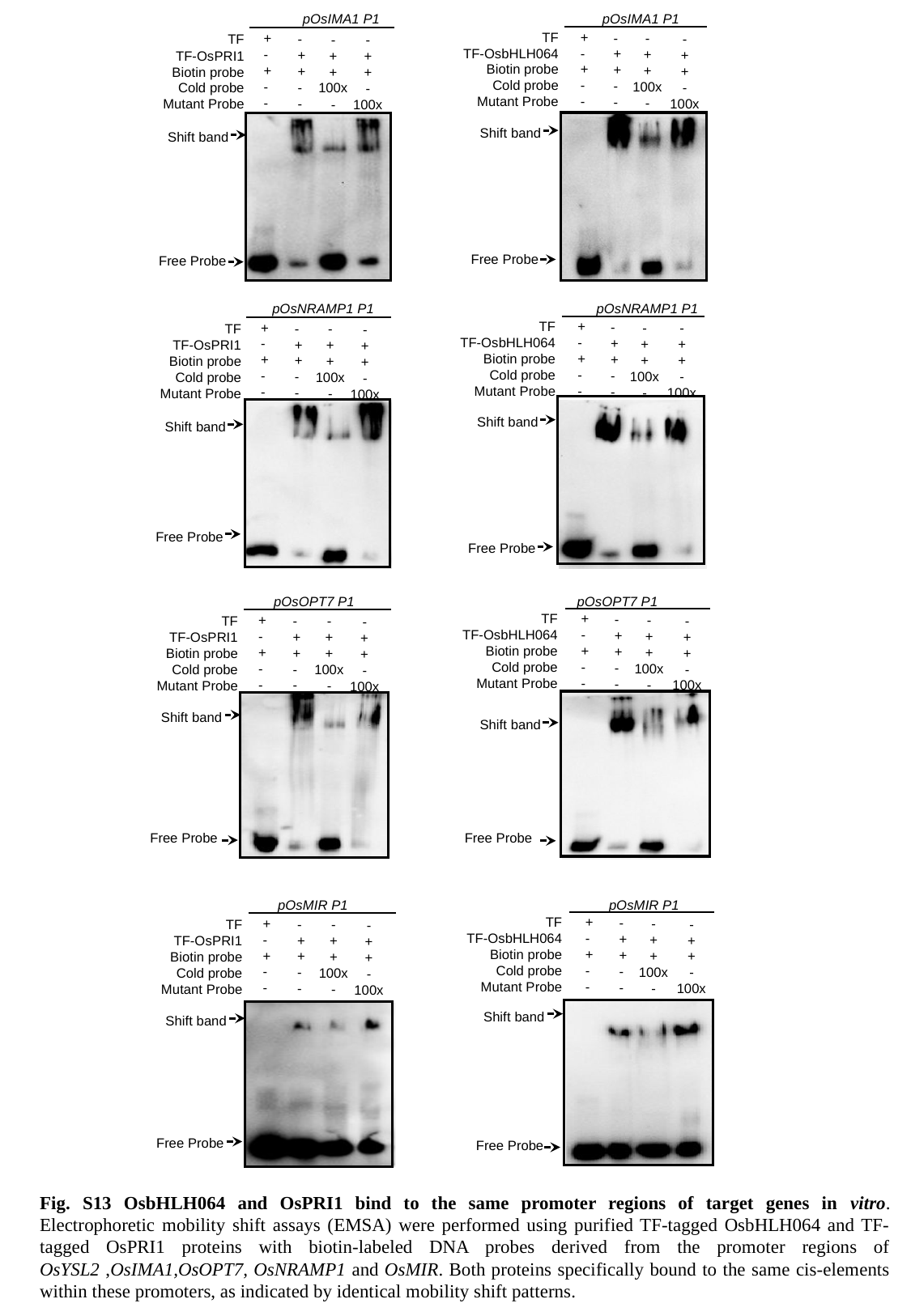

pOsIMA1 P1 pOsIMA1 P1
TF
TF-OsbHLH064
Biotin probe
Cold probe
Mutant Probe
+
-
+
-
-
-
+
+
-
-
-
+
+
100x
-
-
+
+
-
100x
Shift band
Free Probe
+
-
+
-
-
-
+
+
-
-
TF
TF-OsPRI1
Biotin probe
Cold probe
Mutant Probe
-
+
+
100x
-
-
+
+
-
100x
Shift band
Free Probe
pOsNRAMP1 P1 pOsNRAMP1 P1
TF
TF-OsbHLH064
Biotin probe
Cold probe
Mutant Probe
+
-
+
-
-
-
+
+
-
-
-
+
+
100x
-
-
+
+
-
100x
Shift band
Free Probe
+
-
+
-
-
-
+
+
-
-
TF
TF-OsPRI1
Biotin probe
Cold probe
Mutant Probe
-
+
+
100x
-
-
+
+
-
100x
Shift band
Free Probe
pOsOPT7 P1 pOsOPT7 P1
TF
TF-OsbHLH064
Biotin probe
Cold probe
Mutant Probe
+
-
+
-
-
-
+
+
-
-
-
+
+
100x
-
-
+
+
-
100x
Shift band
Free Probe
+
-
+
-
-
-
+
+
-
-
TF
TF-OsPRI1
Biotin probe
Cold probe
Mutant Probe
-
+
+
100x
-
-
+
+
-
100x
Shift band
Free Probe
pOsMIR P1 pOsMIR P1
TF
TF-OsbHLH064
Biotin probe
Cold probe
Mutant Probe
+
-
+
-
-
-
+
+
-
-
-
+
+
100x
-
-
+
+
-
100x
Shift band
Free Probe
+
-
+
-
-
-
+
+
-
-
TF
TF-OsPRI1
Biotin probe
Cold probe
Mutant Probe
-
+
+
100x
-
-
+
+
-
100x
Shift band
Free Probe
Fig. S13 OsbHLH064 and OsPRI1 bind to the same promoter regions of target genes in vitro.
Electrophoretic mobility shift assays (EMSA) were performed using purified TF-tagged OsbHLH064 and TF-tagged OsPRI1 proteins with biotin-labeled DNA probes derived from the promoter regions of OsYSL2 ,OsIMA1,OsOPT7, OsNRAMP1 and OsMIR. Both proteins specifically bound to the same cis-elements within these promoters, as indicated by identical mobility shift patterns.

## Slide 14
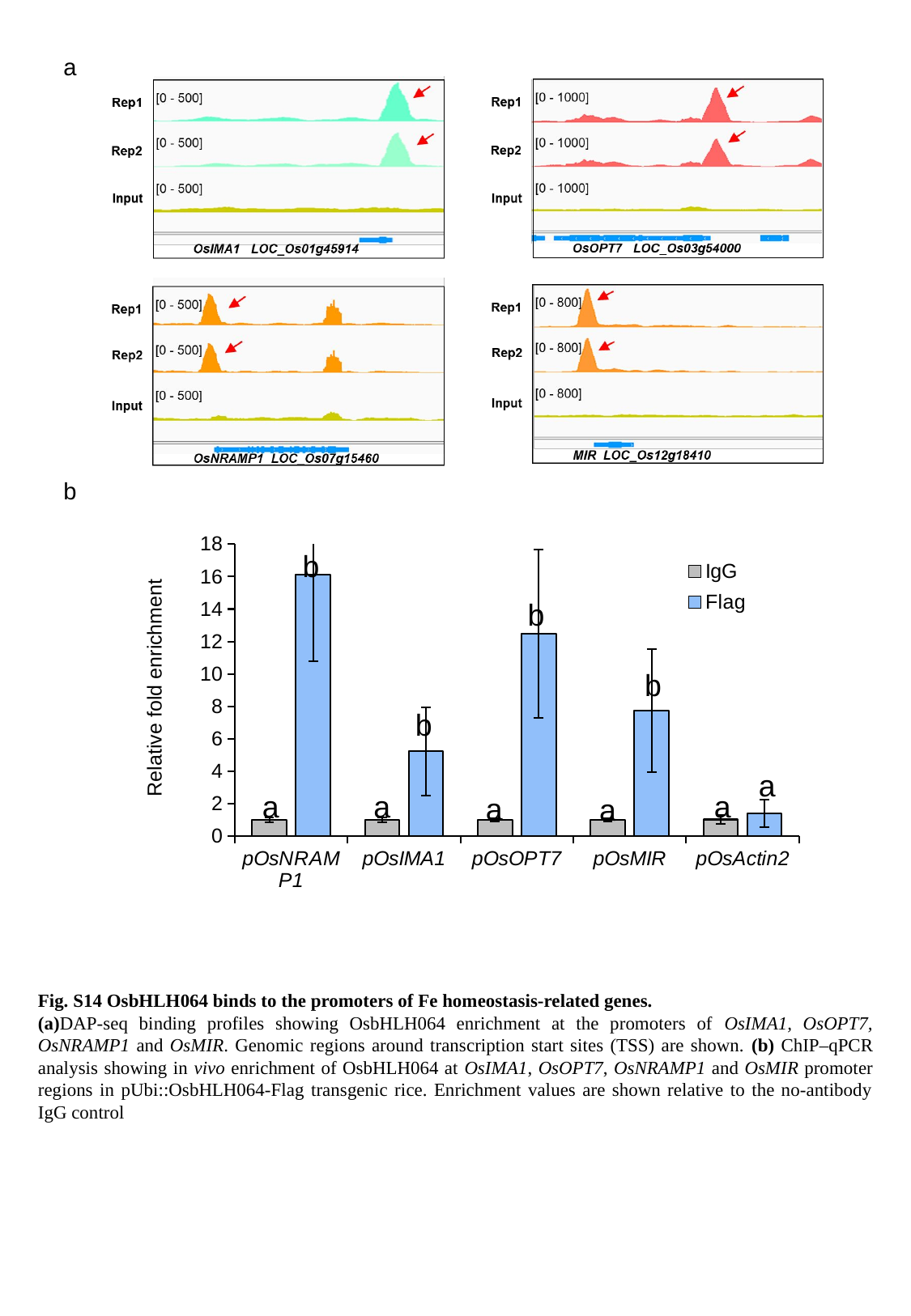

a
b
### Chart
| Category | | |
|---|---|---|
| pOsNRAMP1 | 1.0085961317186556 | 16.09971705159437 |
| pOsIMA1 | 1.0072035357846292 | 5.238789750315955 |
| pOsOPT7 | 1.0031121743087037 | 12.477369189429501 |
| pOsMIR | 1.0022528897433796 | 7.743924578051569 |
| pOsActin2 | 1.0270044737726503 | 1.3985965544855647 |b
b
b
Relative fold enrichment
b
a
a
a
a
a
a
Fig. S14 OsbHLH064 binds to the promoters of Fe homeostasis-related genes.
(a)DAP-seq binding profiles showing OsbHLH064 enrichment at the promoters of OsIMA1, OsOPT7, OsNRAMP1 and OsMIR. Genomic regions around transcription start sites (TSS) are shown. (b) ChIP–qPCR analysis showing in vivo enrichment of OsbHLH064 at OsIMA1, OsOPT7, OsNRAMP1 and OsMIR promoter regions in pUbi::OsbHLH064-Flag transgenic rice. Enrichment values are shown relative to the no-antibody IgG control

## Slide 15
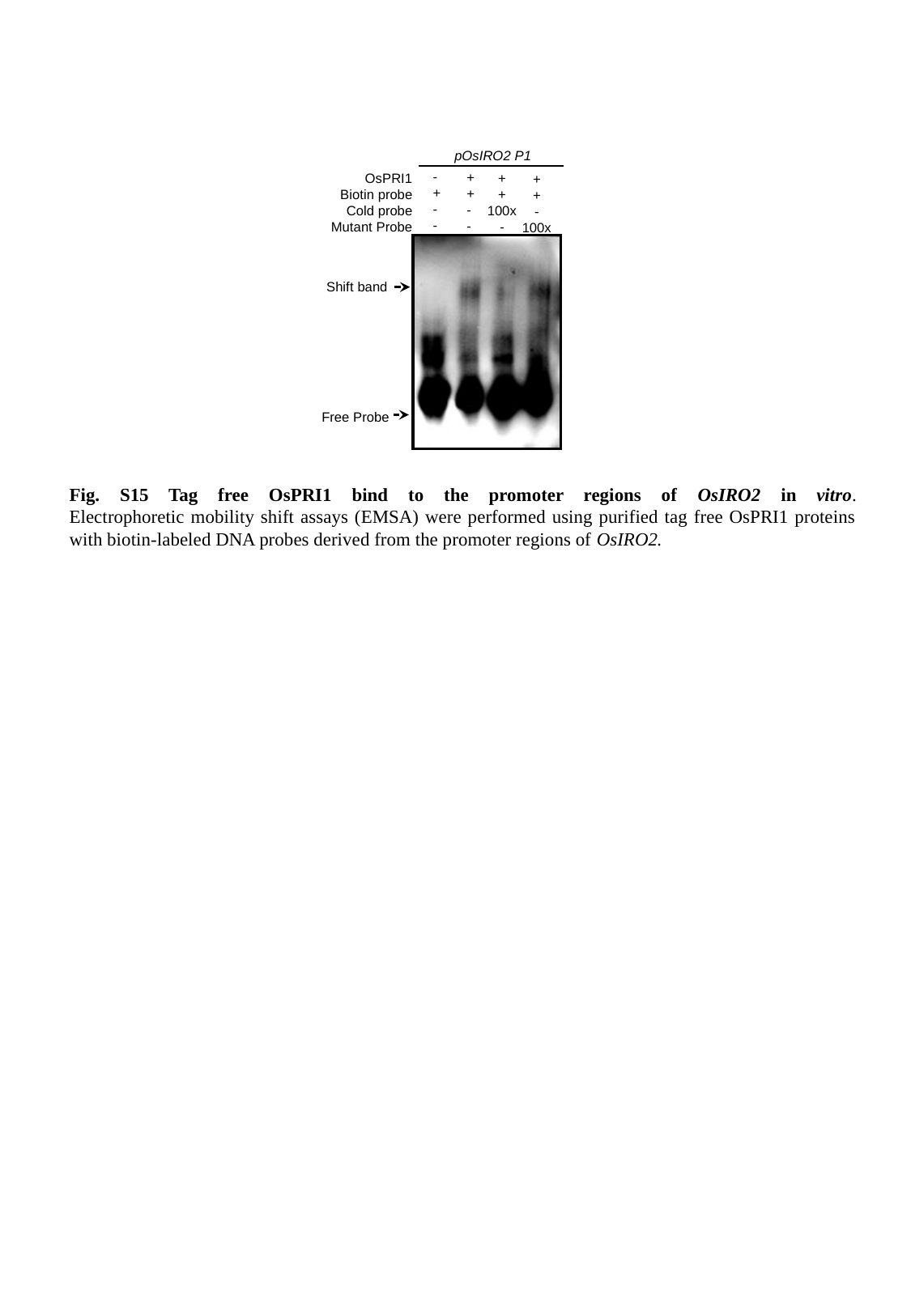

pOsIRO2 P1
-
+
-
-
+
+
-
-
OsPRI1
Biotin probe
Cold probe
Mutant Probe
+
+
100x
-
+
+
-
100x
Shift band
Free Probe
Fig. S15 Tag free OsPRI1 bind to the promoter regions of OsIRO2 in vitro.
Electrophoretic mobility shift assays (EMSA) were performed using purified tag free OsPRI1 proteins with biotin-labeled DNA probes derived from the promoter regions of OsIRO2.

## Slide 16
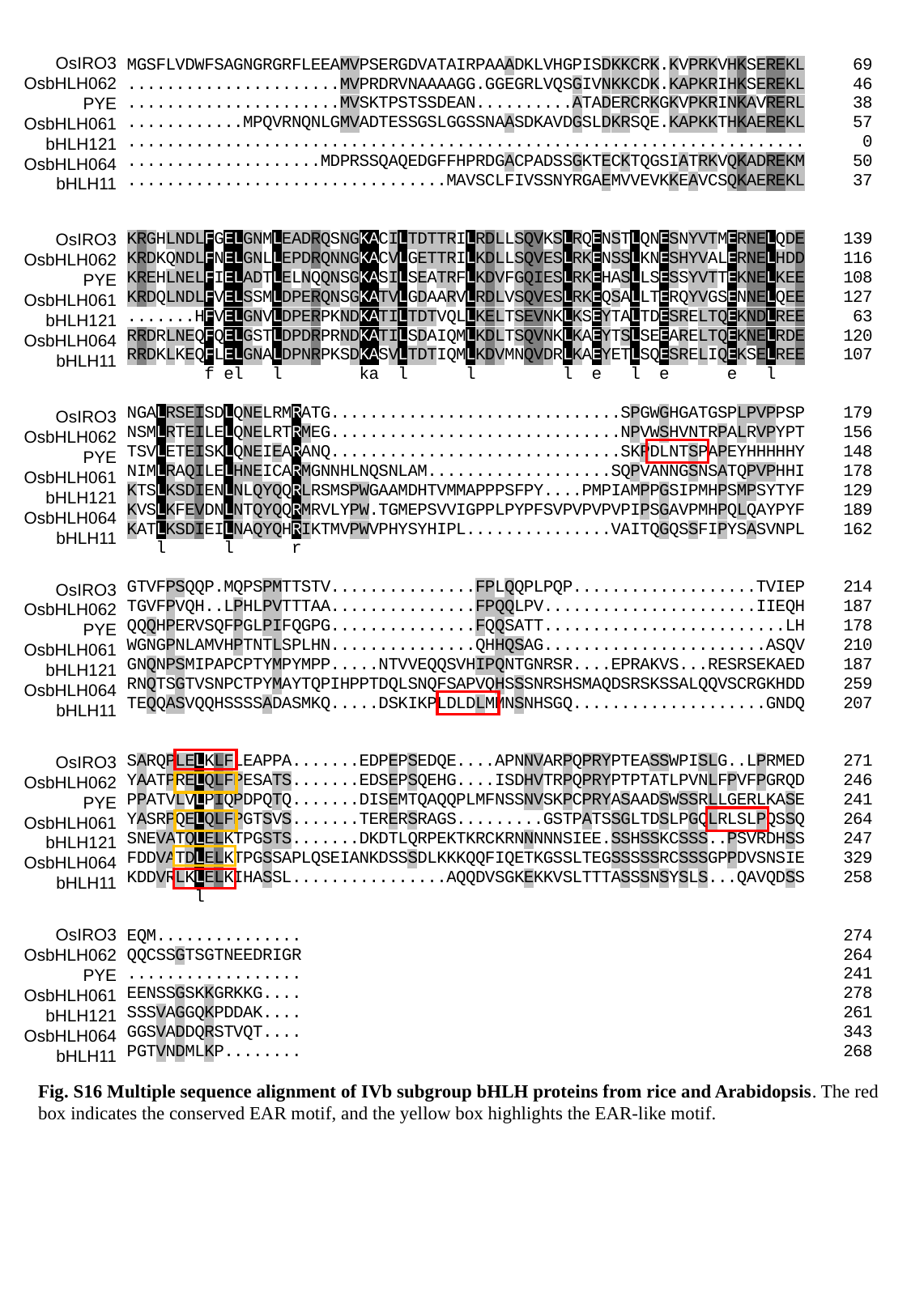

OsIRO3
OsbHLH062
PYE
OsbHLH061
bHLH121
OsbHLH064
bHLH11
OsIRO3
OsbHLH062
PYE
OsbHLH061
bHLH121
OsbHLH064
bHLH11
OsIRO3
OsbHLH062
PYE
OsbHLH061
bHLH121
OsbHLH064
bHLH11
OsIRO3
OsbHLH062
PYE
OsbHLH061
bHLH121
OsbHLH064
bHLH11
OsIRO3
OsbHLH062
PYE
OsbHLH061
bHLH121
OsbHLH064
bHLH11
OsIRO3
OsbHLH062
PYE
OsbHLH061
bHLH121
OsbHLH064
bHLH11
Fig. S16 Multiple sequence alignment of IVb subgroup bHLH proteins from rice and Arabidopsis. The red box indicates the conserved EAR motif, and the yellow box highlights the EAR-like motif.

## Slide 17
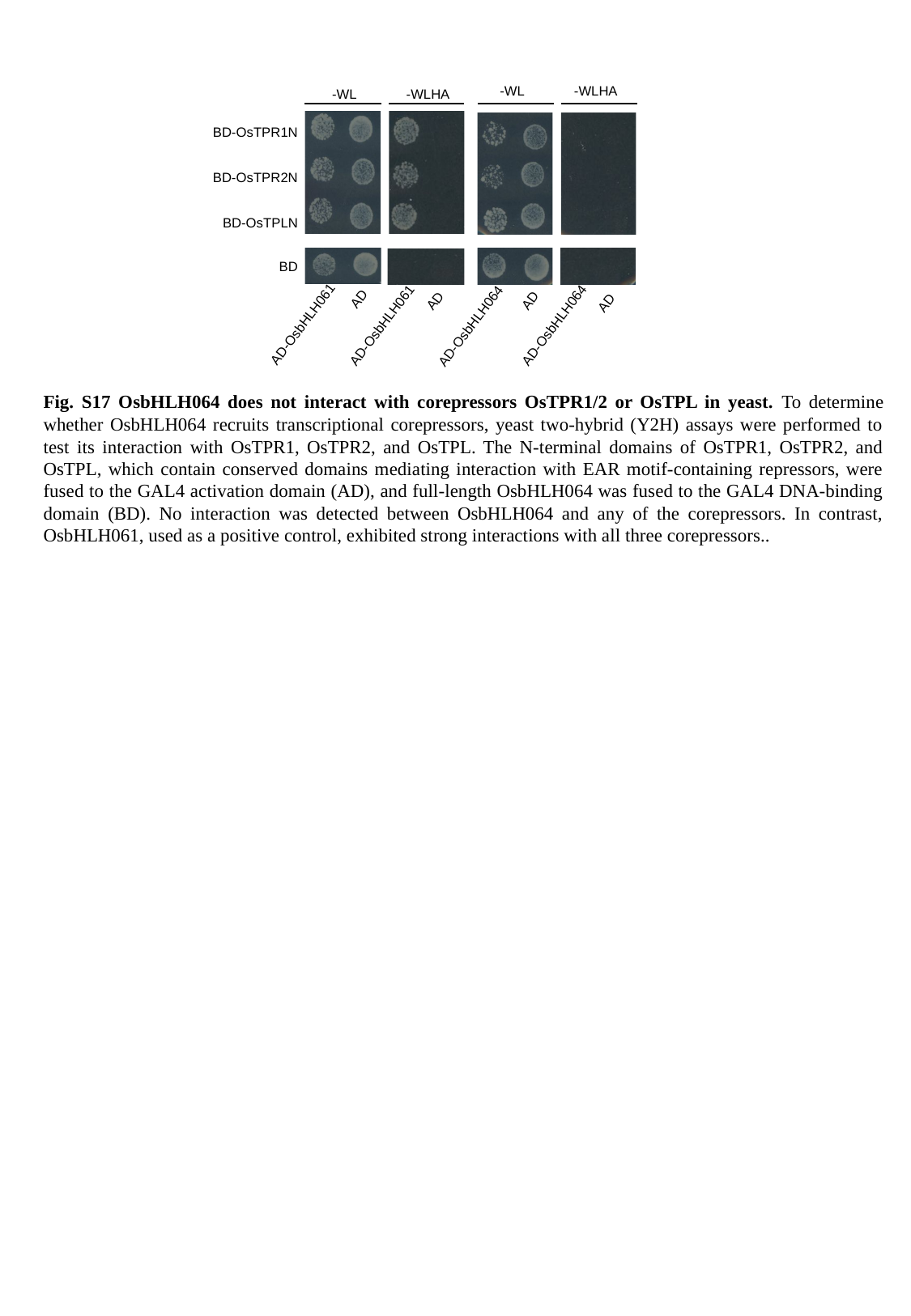

-WL -WLHA
 -WL -WLHA
BD-OsTPR1N
BD-OsTPR2N
BD-OsTPLN
BD
AD
AD
AD
AD
AD-OsbHLH061
AD-OsbHLH064
AD-OsbHLH061
AD-OsbHLH064
Fig. S17 OsbHLH064 does not interact with corepressors OsTPR1/2 or OsTPL in yeast. To determine whether OsbHLH064 recruits transcriptional corepressors, yeast two-hybrid (Y2H) assays were performed to test its interaction with OsTPR1, OsTPR2, and OsTPL. The N-terminal domains of OsTPR1, OsTPR2, and OsTPL, which contain conserved domains mediating interaction with EAR motif-containing repressors, were fused to the GAL4 activation domain (AD), and full-length OsbHLH064 was fused to the GAL4 DNA-binding domain (BD). No interaction was detected between OsbHLH064 and any of the corepressors. In contrast, OsbHLH061, used as a positive control, exhibited strong interactions with all three corepressors..

## Slide 18
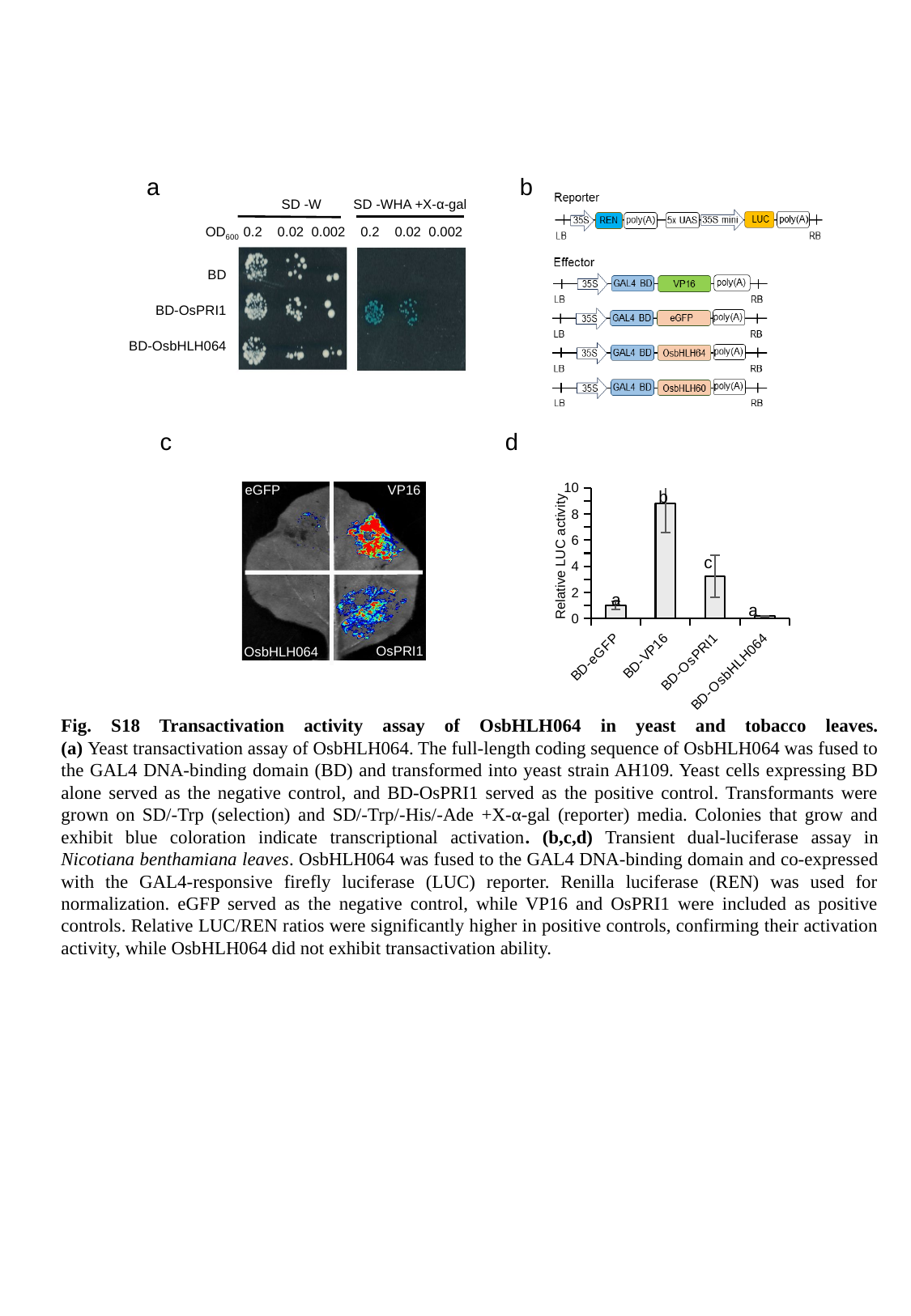

a b
 c d
SD -W
SD -WHA +X-α-gal
OD600 0.2 0.02 0.002 0.2 0.02 0.002
BD
BD-OsPRI1
BD-OsbHLH064
eGFP VP16
OsPRI1
OsbHLH064
### Chart
| Category | |
|---|---|
| BD-eGFP | 1.0 |
| BD-VP16 | 8.80615704380506 |
| BD-OsPRI1 | 3.2518857937911427 |
| BD-OsbHLH064 | 0.165054744492728 |Relative LUC activity
b
c
a
a
Fig. S18 Transactivation activity assay of OsbHLH064 in yeast and tobacco leaves.
(a) Yeast transactivation assay of OsbHLH064. The full-length coding sequence of OsbHLH064 was fused to the GAL4 DNA-binding domain (BD) and transformed into yeast strain AH109. Yeast cells expressing BD alone served as the negative control, and BD-OsPRI1 served as the positive control. Transformants were grown on SD/-Trp (selection) and SD/-Trp/-His/-Ade +X-α-gal (reporter) media. Colonies that grow and exhibit blue coloration indicate transcriptional activation. (b,c,d) Transient dual-luciferase assay in Nicotiana benthamiana leaves. OsbHLH064 was fused to the GAL4 DNA-binding domain and co-expressed with the GAL4-responsive firefly luciferase (LUC) reporter. Renilla luciferase (REN) was used for normalization. eGFP served as the negative control, while VP16 and OsPRI1 were included as positive controls. Relative LUC/REN ratios were significantly higher in positive controls, confirming their activation activity, while OsbHLH064 did not exhibit transactivation ability.
